# Trends in Canadian Suicide Mortality by Age and Sex From 2000 to 2024: Tendances en matière de mortalité attribuable au suicide au Canada selon l’âge et le sexe, de 2000 à 2024

**DOI:** 10.1177/07067437261480922

**Published:** 2026-09-03

**Authors:** Sara Zulyniak, Gina Dimitropoulos, Eric C. Chan, Scott B. Patten

**Affiliations:** 1 Department of Community Health Sciences, 70401 University of Calgary, Calgary, Alberta, Canada; 2 Faculty of Social Work, 2129 University of Calgary, Calgary, Alberta, Canada; 3 Department of Psychiatry, 70401 University of Ottawa, Ottawa, Ontario, Canada; 4 Department of Psychiatry, 2129 University of Calgary, Calgary, Alberta, Canada

**Keywords:** suicide, Canada, adult psychiatry, child and adolescent psychiatry, epidemiology

## Abstract

**Objective:**

Prior research in Canada had only assessed suicide mortality rates through 2018, meaning that suicide trends during and beyond the COVID-19 pandemic are unknown. Thus, this study aimed to extend previous literature by characterizing trends of age- and sex-specific suicide mortality from 2000 to 2024.

**Methods:**

Yearly rates of suicide in Canada were generated from 2000 to 2024 in overall, age- (by five-year age categorizations) and sex-stratified groups. Joinpoint regression analysis was then employed to determine annual percent change (APC) of defined trendlines, and nodes at which the direction or slope of the trendline changed (joinpoints).

**Results:**

Overall rates of suicide in Canada have significantly declined from 2018 to 2024 by an APC of −4.85 (95% CI: −6.26, −3.68; *p* = 0.002). Most male age groups under 40 years of age saw significantly declining trends from 2018 to 2024. For example, males aged 15–19 years had an annual percent decline of 8.37 percentage points from 2018 to 2024 (95% CI: −17.86, −3.52; *p* = 0.01). However, females aged 10 to 14 years saw a significant increase of 2.40 percentage points per year (95%CI: 0.72, 4.39; *p* = 0.007) from 2000 to 2024, while 25–29-year-old females had an annual increase of 1.85 percentage points (95%CI: 1.00, 2.85; *p* < 0.001) during this same time. No other age/sex groups had significantly increasing trends through 2024.

**Conclusions:**

Overall trends and many Canadian age and sex groups have seen declining rates of suicide mortality beginning in 2018, though some young female groups have continued to have increasing rates of suicide from 2000 to 2024. Future literature should look to determine whether these changing suicide trends relate to changing patterns of mental disorder, particularly in young females, or whether other characteristics may better explain the different trends in suicide observed across distinct age and sex groups.

## Plain Language Summary

Trends in Canadian Suicide From 2000 to 2024

The objective of this study was to assess suicide trends in Canada from 2000 to 2024, by age and sex categorizations. Analysis determined the direction and magnitude of trends, along with any notable changes in respective trends. Overall trends and trends in most age and sex categorizations were found to be decreasing from 2018 to 2024, though many groups had increasing trends prior to 2018. There was much variability amongst trends of different age and sex groups. Notably, trends in some young female groups were found to be increasing through to 2024, aligning with previous Canadian evidence. Future research should look to investigate the continued increasing trends of suicide in young Canadian females, along with factors associated with these changing trends.

## Résumé

**Objectif:**

Dans le cadre des recherches menées au Canada par le passé, on avait évalué les taux de mortalité attribuables au suicide jusqu’en 2018 seulement. Autrement dit, les tendances en matière de suicide pendant et après la pandémie de COVID-19 sont inconnues. Ainsi, l’objectif de cette étude était de compléter les données présentées par la littérature existante en caractérisant les tendances en matière de mortalité attribuable au suicide selon l’âge et le sexe de 2000 à 2024.

**Méthodes:**

Les taux annuels de suicide au Canada de 2000 à 2024 ont été obtenus pour l’ensemble des groupes stratifiés selon l’âge (catégories de tranches d’âge de cinq ans) et selon le sexe. Une analyse de régression avec points de rupture a ensuite été utilisée pour déterminer la variation annuelle en pourcentage des courbes de tendance définies et les points de changement auxquels la direction ou la pente de la courbe de tendance a changé (points de rupture).

**Résultats:**

Les taux globaux de suicide au Canada ont considérablement diminué de 2018 à 2024, présentant une variation annuelle en pourcentage de −4,85 (IC à 95% : −6,26 à −3,68; *p* = 0,002). Dans la plupart des groupes d’âge composés d’hommes de moins de 40 ans, on a observé des tendances à la baisse significatives de 2018 à 2024. Par exemple, les hommes âgés de 15 à 19 ans ont présenté une diminution annuelle de 8,37 points de pourcentage de 2018 à 2024 (IC à 95% : −17,86 à −3,52; *p* = 0,01). Toutefois, chez les filles et adolescentes âgées de 10 à 14 ans, on a observé une augmentation significative de 2,40 points de pourcentage par année (IC à 95% : 0,72 à 4,39; *p* = 0,007) de 2000 à 2024, tandis que les femmes de 25 à 29 ans ont présenté une augmentation annuelle de 1,85 point de pourcentage (IC à 95% : 1,00 à 2,85; ^p^ < 0,001) durant la même période. Aucun autre groupe d’âge/de sexe n’a présenté de tendance à la hausse significative jusqu’en 2024.

**Conclusions:**

Les tendances globales, ainsi que de nombreux groupes d’âge et de sexe composés de personnes canadiennes, ont présenté une diminution des taux de mortalité attribuable au suicide à partir de 2018, bien qu’on ait constaté une augmentation continue des taux de suicide de 2000 à 2024 dans certains groupes de jeunes femmes. Les publications à venir devraient chercher à déterminer si ces tendances changeantes en matière de suicide sont liées à l’évolution des profils de troubles mentaux, en particulier chez les jeunes femmes, ou si d’autres caractéristiques peuvent mieux expliquer les différentes tendances en matière de suicide observées entre les groupes d’âge et de sexe.

## Background

Previous Canadian suicide analyses have observed overall decreases in age-standardized mortality rates by suicide from 1980 to 2017, though many have identified differential trends by age and sex categorizations.^1–3^ Primarily, suicide trends in Canada appear to be relatively stable, or declining in all age and sex categorizations except adolescent and young adult females, who have seen increases in their rates of suicide through 2018.^3,4^ Age and sex differences have similarly been identified by other global analyses, with an Austrian study showing declines in young male suicide from 2001 to 2014, though young female suicide trends remained stable over this time.^5^ On the contrary, suicide in adolescents of both sexes in the United States appears to be increasing from 1999 to 2020, though the degree of increase varied by ethnicity and method.^6^ While young people, and more specifically young females, have seen recent increases in their rates of suicide mortality, middle-aged adult males continue to have the highest age-specific rate of suicide in Canada, up to three to four times greater than any rates observed in young females from 2024 mortality data.^7^ Thus, suicide in middle-aged and older adults in Canada continues to be an important topic of research attention, despite some evidence of changing trends in young females.

Looking to evidence on other suicide-related behaviours, it is important to note that suicide attempts, self-harm, and suicidal ideation represent distinct concepts that are often conflated within discussions on suicide. Self-harm can occur without the intent of suicide, while suicide attempts are considered actions performed with the intention of suicide.^8^ In most literature, suicidality is an umbrella term often encompassing all of suicide attempts, self-harm, and suicidal ideation, serving as a general descriptor of one or more of these characteristics.^9^

The previously detailed suicide mortality analyses are supported by evidence that self-reported suicide attempts and suicidal ideation were increasing in young Canadian females prior to the COVID-19 pandemic.^10^ Of some concern is whether this trend has continued throughout COVID-19. Broadly, research has indicated that self-harm related emergency visits increased in 2020 when compared to data from 2018 and 2019.^11^ Further, when assessing paediatric mental health-related emergency department visits temporally over COVID-19, the largest increase in visits by both sexes was seen during initial school closures, indicating that routine disruptions and feelings of isolation or loneliness associated with the beginning of the pandemic may have driven an increase in poor mental health.^12^ Further, an Ontario cross-sectional analysis of adolescents described risk factors associated with suicidal ideation, such as low perceived social support, worthlessness, low self-esteem, and cannabis use, which could have been amplified during COVID-19.^13^ Much of the suicide and suicidality trend evidence, though, saw a continuation of sex-specific trends. Notably, an assessment of hospitalizations and emergency department visits for self-harm using Canadian administrative data found that females aged 10 to 19 years had higher than expected rates of self-harm in parts of 2020 and 2021, when compared to other age and sex groups that experienced minimal fluctuations.^14^ Similarly, this finding was replicated by several time-series analyses that identified significant increases in the rate of suicidal ideation and self-harm hospitalizations in young females during the pandemic, when compared to pre-pandemic years.^15,16^

Many of these prior analyses focussed on adolescent or young adult samples, meaning recent trends in suicide among middle-aged/older Canadian adults during and beyond the COVID-19 pandemic are relatively unknown. Further, the latest Canadian suicide trend analyses have not reported on data beyond 2018, and literature on other suicidal behaviour has been primarily focussed on the acute phases of COVID-19, making it difficult to assess the current landscape of suicide in Canada. Additionally, no Canadian suicide analyses have assessed mortality data beyond COVID-19 in quantifying whether trends may have changed following such an impactful global health event. The broad reassessment of suicide trends in Canada by age and sex, using the most recent Canadian mortality data, may be necessary to fill some of these knowledge gaps. Thus, this analysis aims to describe the current trends in Canadian suicide mortality by age and sex categorizations from 2000 to 2024.

## Methods

The number of deaths by suicide in Canada from 2000 to 2024 were identified using Statistics Canada Table 13-10-0392-01,^17^ specifying deaths by intentional self-harm as coded by ICD-10 codes X60 to X84 and Y87.0. These deaths were assessed in all ages and by sex-stratification (male and female), as well as stratified by 5-year age categories (i.e., 10-14, 15-19, to a maximum age group of 85+) and sex. Mid-year population counts from 2000 to 2024 were used to generate cause-specific mortality rates by suicide in each group, as identified by Statistics Canada Table 17-10-0005-01, and standard errors of these rates were calculated.^18^ Notably, mid-year population counts are now provided by gender, specified as men + and women+, while cause-specific mortality is provided by sex. There may be discrepancies between those who identify as transgender, non-binary, or other gender non-conforming individuals in mortality and mid-year population counts; however, there is no ability to link data from these aggregated tables, ultimately lacking any individual identification necessary to account for potential differences. Canadian census data from 2021 has reported that 0.33% of the population over age 15 identified as transgender or non-binary, while this prevalence is greater in young adults aged 20–24 at 1%.^19,20^ Further, research in other high-income countries has found elevated rates of suicide mortality in transgender individuals when compared to non-transgender people, highlighting an increased risk in this population.^21^ Despite this increased risk of suicide, and concentration of gender diversity in young people, the small percentage of differential sex and gender classification between the two data sources is unlikely to result in substantial bias of the calculated mortality estimates.

Joinpoint regression analysis of overall, age- and sex-stratified suicide mortality rates was completed using the SEER*Stat software developed by the National Cancer Institute of the National Institutes of Health (NIH); Division of Cancer Control and Population Sciences.^22,23^ Joinpoint regression is a type of linear spline regression that uses permutation tests to determine knots (joinpoints) between possible changing trend patterns amongst a set of estimates over time, aiming to fit the simplest possible model.^24,25^ The joinpoint model calculates an annual percent change (APC) of each identified trend section and determines intercept and slope value statistical significance, accounting for multiple tests with the Bonferroni correction. The grid-search method of joinpoint regression was specified,^26^ and log-linear models with weighted BIC selection methods were employed for regression analysis of each age and sex group. A maximum of four joinpoints was specified for all models given the algorithmic recommendations of the software relative to the number of suicide rates for each group.^22^

## Results

*Overall and sex-stratified trends:* When considering overall trends in all ages and both sexes, there was a significantly increasing APC of 1.09 (95% CI: 0.56, 3.08; *p* = 0.007) percentage points from 2007 to 2018, though from 2018 to 2024 the suicide rate decreased by −4.83 (95% CI: −6.26, −3.68; *p* = 0.002) percentage points per year (joinpoint graph in Supplemental Appendix A, Figure A.1; APC metrics in Supplemental Appendix E, Table E.1). This trend pattern was similarly observed in males of all ages, though females saw greater increases from 2012 to 2016, with an APC of 3.94 (95% CI: 1.41, 6.36; *p* = 0.030) percentage points, but also had a significantly decreasing trend of annual suicide rate from 2016 to 2024 (sex-stratified graphs are located in Supplemental Appendix A, Figure A.2 and Figure A.3, while APC metrics can be found in Supplemental Appendix E, Table E.1).

**Figure 1.**
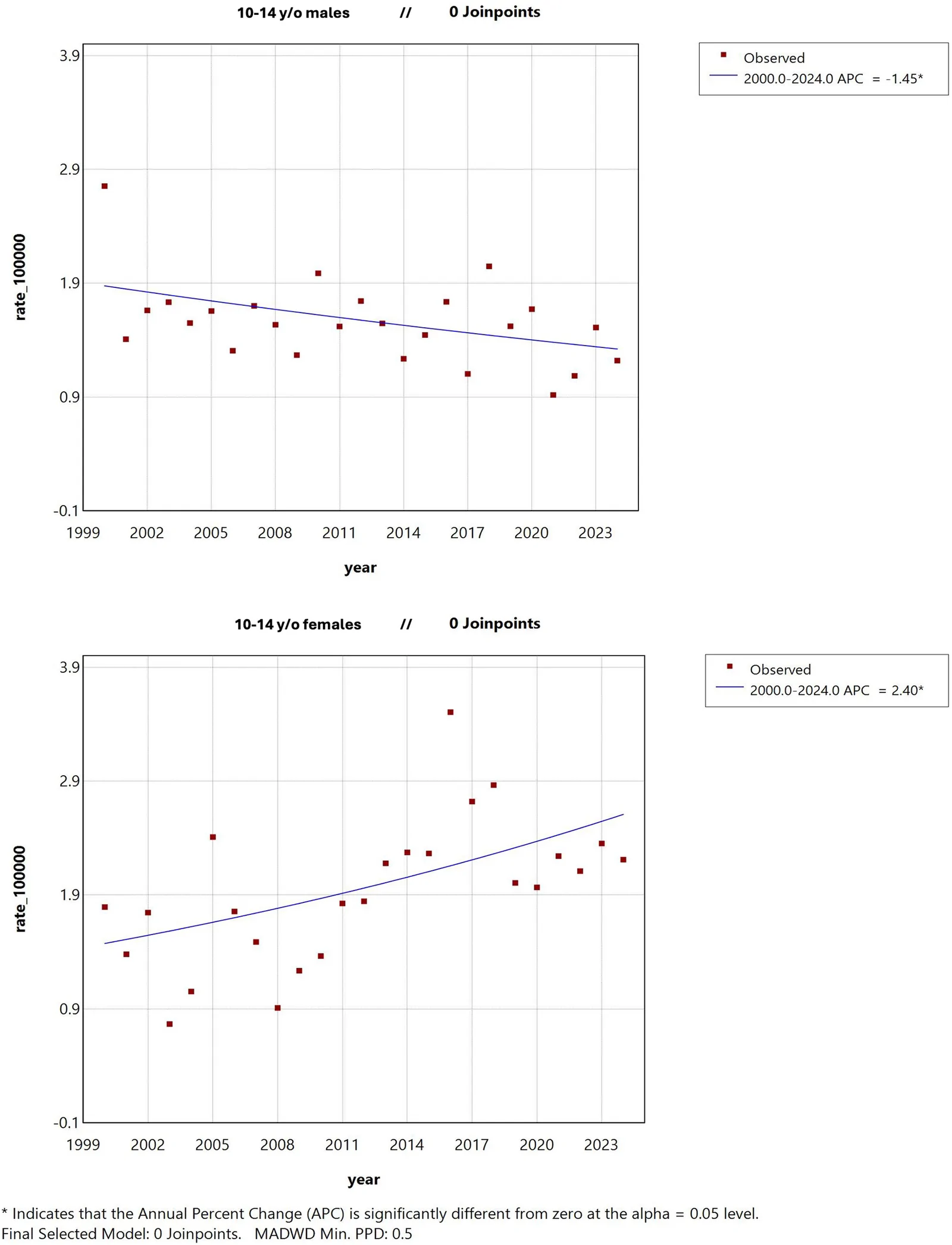
Annual percentage change (APC) of age between 10 and 14.

**Figure 2.**
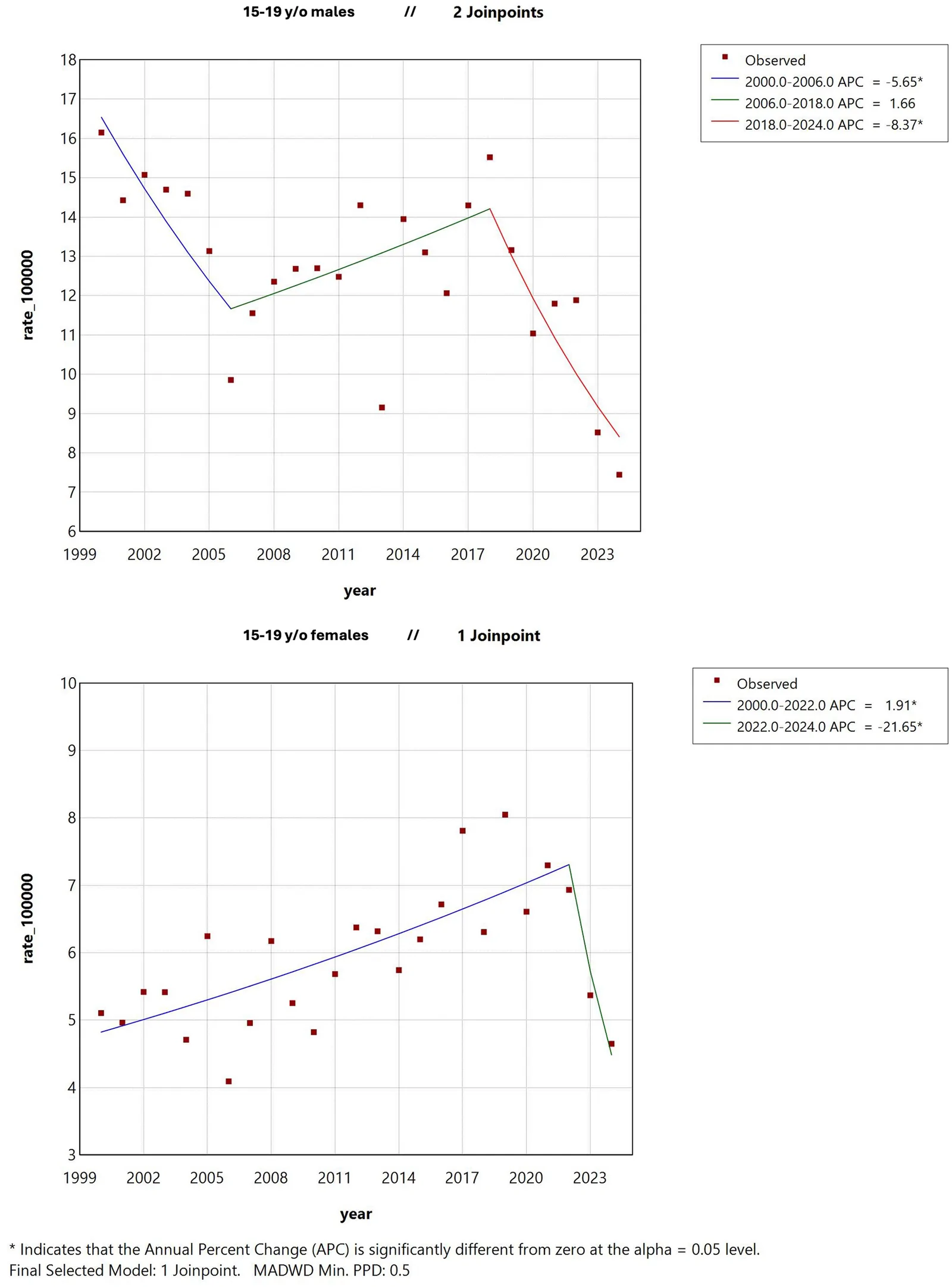
Annual percentage change (APC) of age between 15 and 19.

**Figure 3.**
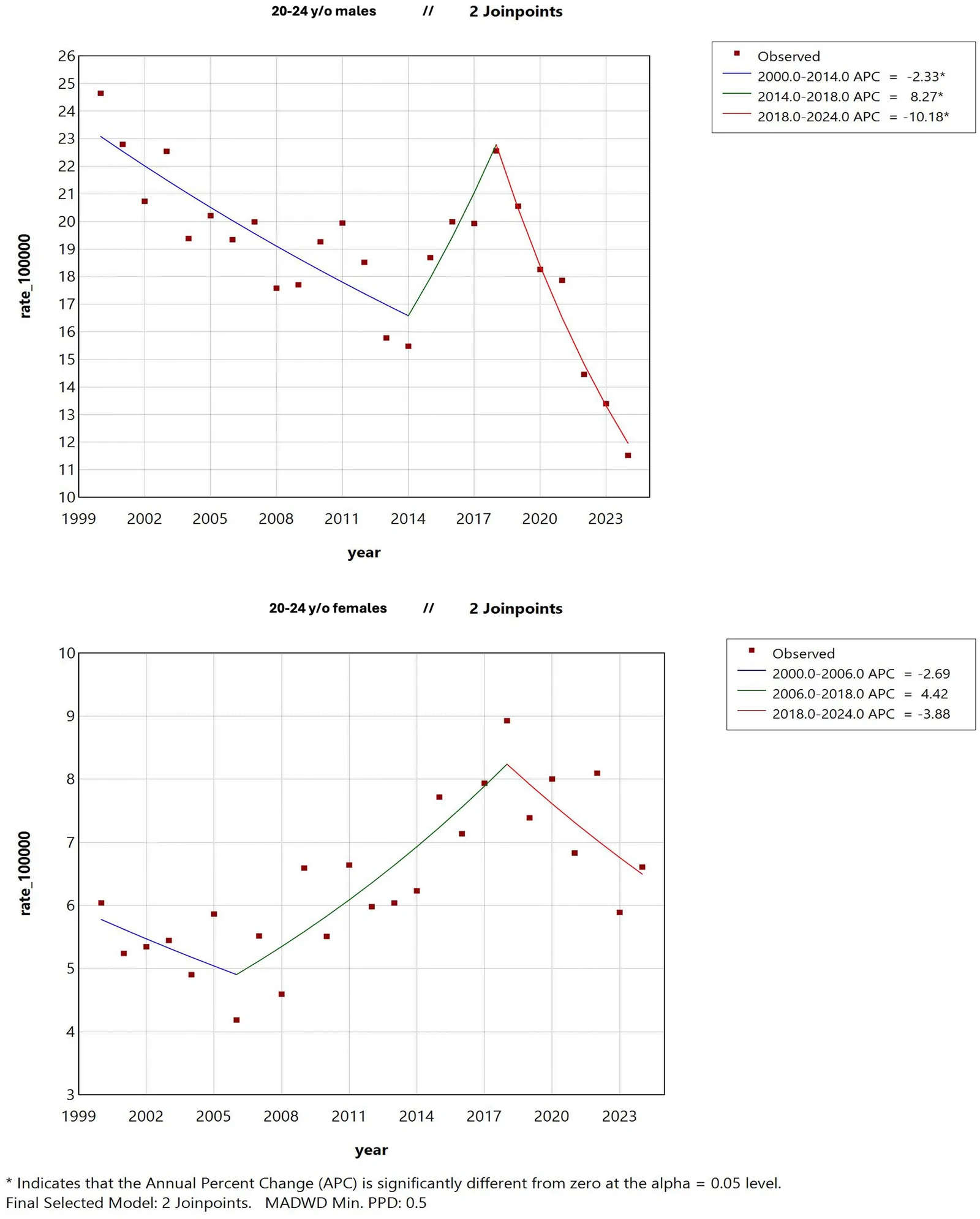
Annual percentage change (APC) of age between 20 and 24.

**Figure 4.**
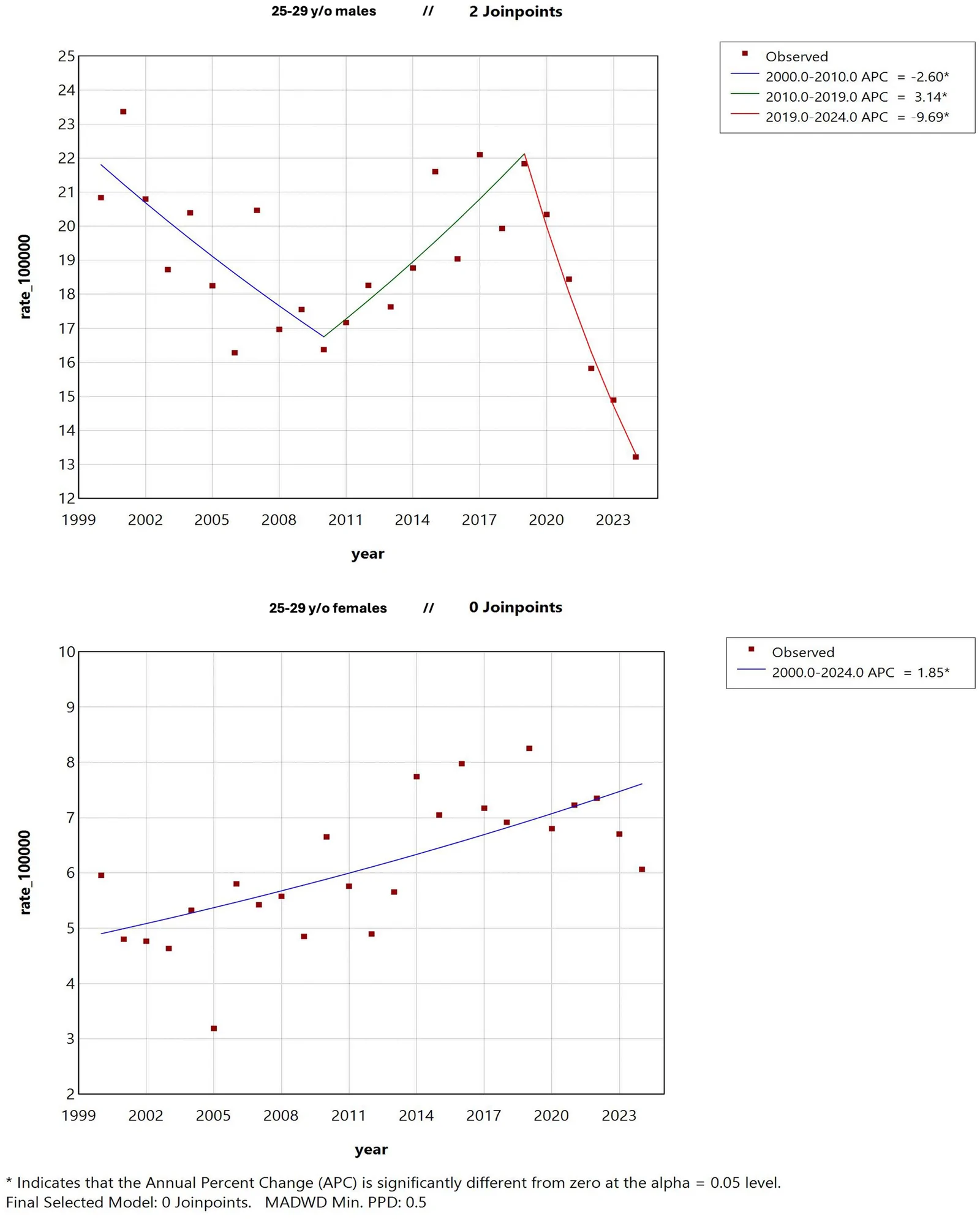
Annual percentage change (APC) of age between 25 and 29.

**Figure 5.**
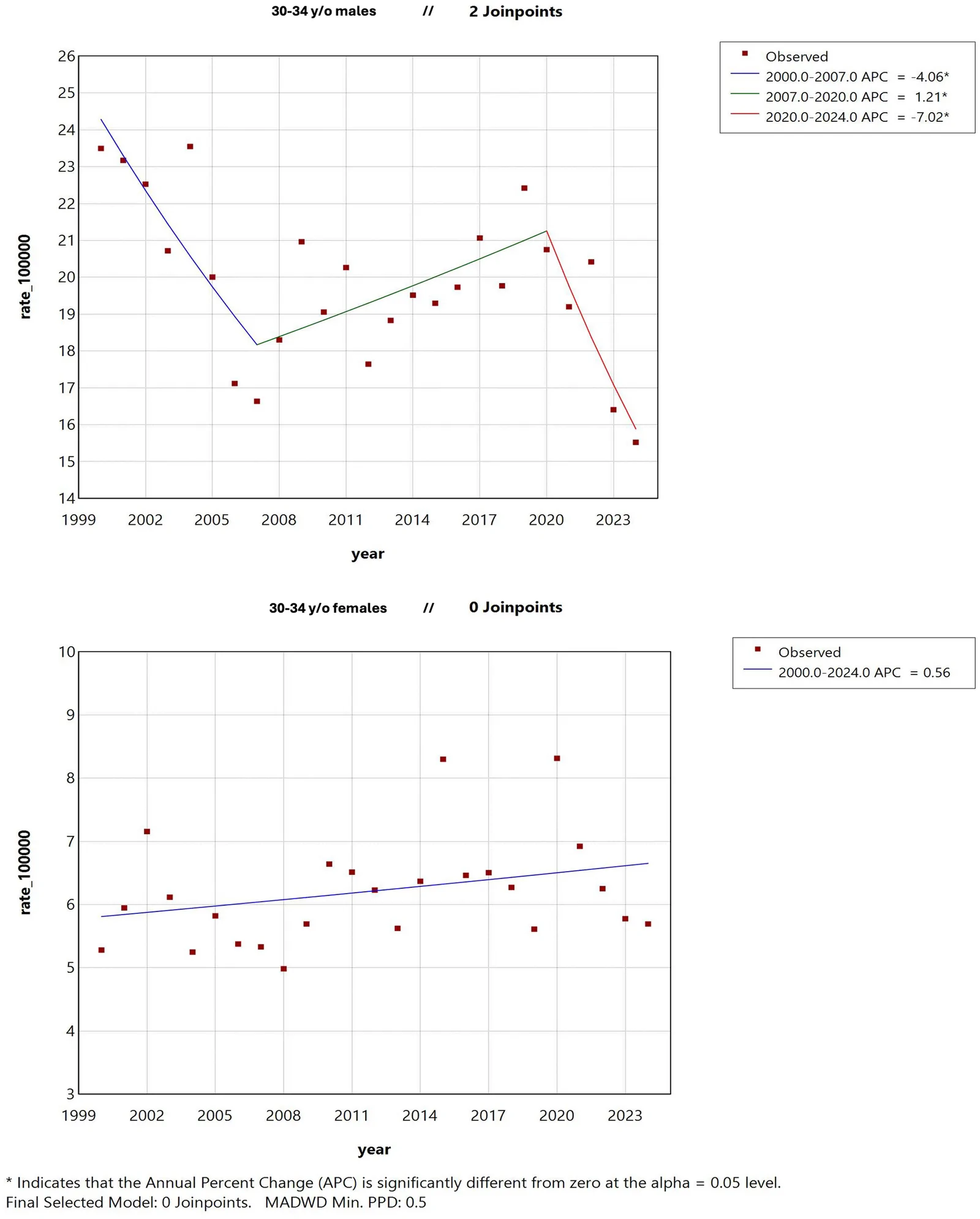
Annual percentage change (APC) of age between 30 and 34.

*Age-stratified males*: As seen in Figure 1, males aged 10 to 14 years saw their rate of suicide decrease from 2.75 per 100,000 in 2000 to 1.22 per 100,000 in 2024, with an APC of −1.45 (95% CI: −2.91, −0.05; *p* = 0.043). In the 15- to 19-year-old male group of Figure 2, however, the suicide rate ranged from 16.15 per 100,000 in 2000 to 9.86 per 100,000 in 2006 (APC_2000–2006_ = −5.65; 95% CI: −17.02, −1.38; *p* = 0.01), then to 15.52 per 100,000 in 2018 (APC_2006–2018_ = 1.66; 95% CI: −0.03, 12.43; *p* = 0.05), and finally to a minimum rate value of 7.44 per 100,000 observed in 2024 (APC_2018–2024_ = −8.37 95% CI: −17.86, −3.52; *p* = 0.01). Other male groups under 40 years of age mirrored trends seen in those 15–19 years of age, as seen in Figures 3 to 5, while additional graphs of older age groups can be found in Supplemental Appendix B.

Males aged 40 to 44 years saw a decrease of −1.19% per year (95% CI: −1.68, −0.73; *p* < 0.001) from 2000 to 2024 (Supplemental Appendix B, Figure B.3), though some older age groups had increasing trends of suicide in earlier years. For example, 55–59-year-old males had a significantly increasing APC of 1.24 from 2000 to 2018 (95%CI: 0.65, 2.30; *p* < 0.001), but saw a reversal of this trend from 2018 to 2024 (APC = −4.16; 95%CI: −10.10, −1.53; *p* = 0.003), as seen in Supplemental Appendix B, Figure B.9. Most male groups aged 60 years and older saw similar patterns, with decreasing trends around 2017. There was much variability in suicide rates amongst the oldest males aged 85 +  years, as per Figure B.19 in Supplemental Appendix B, and the generally small counts of suicide in these oldest age groups are shown in Supplemental Appendix C.

*Age-stratified females*: Young females showed different patterns of suicide mortality when compared to males of the same age groups. Despite 15- to 19-year-old females having increasing rates of suicide from 2000 to 2022 (APC=1.91; 95%CI: 1.24, 3.03; *p* > 0.001), their most recent rates were decreasing from 2022 to 2024 (APC = −21.65; 95%CI: −33.41, −3.91; *p* = 0.016), as seen in Figure 2. Similar changing trends were observed in 20–24-year-old females (Figure 3), with increases from 2006 to 2018 (APC=4.42; 95%CI: −6.78, 19.39; *p* = 0.061) and decreases from 2018 to 2024 (APC = −3.88; 95%CI: −14.07, 3.05; *p* = 0.126), though this recent decreasing APC was not statistically significant. Interestingly, both 10–14-year-old (APC=2.40; 95%CI: 0.72, 4.39; *p* = 0.007) and 25–29-year-old (APC=1.85; 95%CI: 1.00, 2.85; *p* < 0.001) females had continuously increasing rates of suicide through 2024, as shown in Figures 1 and 4, respectively. However, the small numbers of suicides in the youngest female age groups may have contributed to increased variability and reduced precision of these estimates, as seen in Supplemental Appendix D. Looking to older age groups, 30- to 34-year-old females also showed increasing rates of suicide through 2024 (APC=0.56; 95%CI: −0.28, 1.49; *p* = 0.169), though the APC was not statistically significant (Figure 5). Female groups between ages 40 and 64 years had either unchanging or increasing rates of suicide in initial years, but generally showed recent decreases in suicide through 2024 (Supplemental Appendix B). Notably, 50–54-year-old females had a positive slope from 2000 to 2018 (APC=0.89; 95%CI: 0.002, 2.33; *p* = 0.05), but a negative slope from 2018 to 2024 (APC = −8.45; 95%CI: −18.02, −4.16; *p* < 0.001), as seen in Supplemental Appendix B, Figure B.8. Similar patterns were observed in female groups up to 65 years of age, beyond which no significant APC trends were identified through 2024 (age- and sex-specific APC values can be found in Supplemental Appendix E, Table E.2).

## Discussion

Overall, age- and sex-stratified groups in Canada have seen varying trends in their cause-specific suicide mortality rate from 2000 to 2024, with declining trends in the majority of groups beginning around 2018. Interestingly, there were no notable increases in overall, or age- and sex-specific suicide rates during the peak years of the COVID-19 pandemic in 2020 and 2021, supporting some early global evidence from the pandemic.^27–29^ The broad inclusion of available mortality data, including estimates from COVID-19 and most recent 2024 estimates, expands upon previous Canadian suicide analyses that only captured data up to 2018.^3^ Despite the recent declines observed in a majority of stratifications, some young female groups (ages 10 to 14, and 25 to 29) continued to see increasing suicide rates from 2000 to 2024, similar to trends identified by previous analyses.^3,4,30^ Though 25–29-year-old females saw decreasing suicide rates from 2022 to 2024, the youngest (10–14-year olds) female trend shows no such indications of decline. However, the number of deaths by suicide in 10–14-year-old females is much smaller when compared to other age groups, so there is a greater potential for increased variability and reduced precision of these estimates. Regardless, the continued increasing suicide rates in some young females identified by this analysis align with previous literature in highlighting a worrisome trend, and may be important for further investigation.

Looking to factors that may be contributing to these observed trends in suicide, there is a great deal of heterogeneity in the literature regarding specific risk factors for suicide. Some of this heterogeneity may relate to age differences in suicide risk factors, as one analysis identified childhood maltreatment to be most associated with suicide attempt in those under 18 years of age, while substance abuse and mood disorders were most strongly associated with suicide attempt in those over 35 years of age.^31^ However, age differences may not be solely contributing to the heterogeneity of suicide risk prediction, as a meta-analysis of longitudinal studies in adolescents and young adults identified differential risk factors between males and females of this specific age group.^32^ For example, the strongest predictors of suicide attempt in young females included eating disorder, dating violence and interpersonal problems, whereas prominent male predictors included conduct issues, parental separation, and access to means.^32^ Though specific mental disorders most associated with suicidal behaviour may vary by both age and sex, meta-analytic evidence has continuously reaffirmed that any diagnosed mental disorder is strongly associated with suicide and suicidal behaviour.^33,34^ Recent trend analyses of Canadian community samples have identified the most prominent increases in poor mental health to have been amongst adolescent and young adult females,^10,35^ pointing to a potential hypothesis explaining why suicide rates in this group have continued to increase. However, the prevalence of mental disorders is not exclusively increasing in young Canadian females, with young males also seeing an increasing prevalence of poor mental health, along with middle-aged/older adults of both sexes, despite suicide declining in these age and sex groups.^35^ Given these complications, other factors may better explain these changing trends of suicide amongst certain age and sex groups.

It is important to note that existing mental disorder may not always be the best predictor of suicide. In fact, one longitudinal study from the US suggested that up to 20% of people with no preceding psychiatric diagnosis had attempted suicide in their lifetime.^36^ In a Canadian context, previous evidence has demonstrated that up to two-thirds of Canadians reporting suicidality did not also have depression, though individuals reporting only suicidal behaviour were also less likely to have had any contact with mental health service providers, meaning this suicidality is more likely to have gone undetected.^37^ The apparent discrepancy of current trend patterns suggests suicide and common mental disorders, such as depression, may be less strongly associated over time, but this relationship could differ by age and sex. Distinct assessment of suicide-related behaviour as a symptom of another comorbid mental disorder (such as major depression, for example), or as an independent occurrence, is important for future literature on this subject. Most likely, a complex array of factors are contributing to the changing rates of suicide seen by this analysis, though the makeup of these factors may be changing over time, and may differ by age and sex categorizations. Many complex theories exist that attempt to explain suicidal behaviour, such as those related to specific characteristics common to those who are physically capable of attempting suicide.^38–40^ Careful future analysis considering these nuanced factors, alongside age and sex differences, may be required to determine the most relevant predictors of suicide trends, particularly if increasing rates of suicide in young females are continuously observed.

Beyond the identification of potential risk factors for suicide, other literature has focussed on the distinct suicidal behaviours between certain age and sex groups. Recent Canadian evidence has demonstrated that though females have higher rates of suicide attempts, males continue to have much greater rates of suicide mortality; a relevant distinction when considering the importance of sex-specific suicide prevention measures.^41^ Commonly, these sex differences have been attributed to males employing more lethal means of suicide,^42^ though patterns of suicide means may be changing. Some evidence has highlighted that while rates of suicide by poisoning and firearms in men are decreasing, suicides attributable to hanging are increasing from 1980 to 2018.^2,43^ Changing patterns in the means of suicide could be contributing to the changes observed in suicide mortality trends, though suffocation is often considered to be a more lethal means than poisoning. Further, suicide by suffocation or hanging has been explored by qualitative literature, and described by survivors of a previous near-fatal suicide attempt to be “easy, quick, clean and simple,”^44^ rendering a perceived difficulty in preventing these deaths. Considering the increased use of suffocation and hanging identified by previous literature, future research should look to characterize the most prevalent means of suicide from 2018 to 2024, as many age and sex groups had observed an overall decreasing pattern of suicide during this time. If the prevalence of suffocation and hanging continues to increase, while other methods of suicide are in decline, this may be problematic given the relative difficulty of prevention measures specific to this mechanism when compared to those for suicide by firearms and poisonings, for example. Thus, it is important for future literature to consider, or stratify by the means of suicide in attempting to explain the observed changes in overarching suicide trends.

When considering means of suicide, there is always an additional risk of the misclassification of suicide deaths in population-level mortality data, particularly when it comes to poisoning. Previous evidence has noted the increase of unintentional and undetermined poisoning deaths alongside decreasing rates of suicides labelled as poisonings, calling the accuracy of this reporting into question.^45^ There is a large body of global literature that has examined the misclassification of suicides, some of this evidence pointing to the discrepancies in autopsy performance and incomplete cause-of-death reporting to be primary drivers of this potential misclassification.^46,47^ Regardless, Canadian evidence considering some of these commonly misclassified categories of mortality has found minimal differences in the overall interpretations of suicide mortality trends.^48^ Additionally, a Scandinavian study retrospectively reclassifying deaths of accidental or undetermined intent found very few actual misclassified suicides, easing some widespread concern.^49^ Though it still remains to be considered that the declining rates of suicide by poisoning seen in recent literature could be a misclassification issue, this misclassification is unlikely to impact broad population-level mortality analyses, as presented here.

Notably, many age groups aged 45 +  experienced a greater decrease in the rate of suicide mortality around 2017, primarily in males. Though it is not possible to assess the impacts of Medical Assistance in Dying (MAID) legislation, which was allowable in Canada in 2016, over 95% of deaths by MAID have occurred in those over 45 years of age (though the majority of MAID recipients are female).^50^ It is important for the continual comparison of deaths by MAID and suicide that ICD-10 coding for MAID be developed, supporting efforts to understand how this legislation may be impacting suicide mortality in Canada.

Limitations of this study include the previously discussed potential for misclassification of suicide as accidents or poisonings of undetermined intent, leading to possible under-estimation of the true number of suicide deaths in Canada.^45^ There is also potential for over-estimation of deaths by suicide considering the recent epidemic of opioid-related overdose deaths in Canada that may have been misclassified as suicides.^51^ Further, since suspected suicides and accidental deaths often require extensive review by coroners and medical examiners, particularly in young people, the most recent Statistics Canada mortality data may be updated upon completion of these reviews, meaning there may also be an under-estimation of suicide mortality in 2024.^52^ Many of the youngest age groups, particularly in females, had few counts of annual suicide, subjecting these estimates to reduced precision and increased variability of associated trends. Additional individual-level information, such as prior mental disorder diagnoses or histories of abuse, was not able to be assessed alongside suicide mortality rates given the use of publicly available aggregate data, though these were previously detailed to be important factors related to suicide.

## Conclusion

Most age and sex groupings in Canada have seen decreases in their rates of suicide mortality beginning in 2018, though some young female groups have seen continued increases in suicide from 2000 to 2024. Future literature should look to identify characteristics that may be associated with these changing trends in suicide, while continuing to emphasize differences between unique age and sex categorizations, as substantial literature has highlighted the importance of considering these basic demographic factors. Additionally, the examination of comorbid mental disorder diagnoses, or lack thereof, relative to individual occurrences of suicidal behaviour may help to further understand future trends.

## Supplemental Material

sj-docx-1-cpa-10.1177_07067437261480922 - Supplemental material for Trends in Canadian Suicide Mortality by Age and Sex From 2000 to 2024: Tendances en matière de mortalité attribuable au suicide au Canada selon l’âge et le sexe, de 2000 à 2024 Supplemental material, sj-docx-1-cpa-10.1177_07067437261480922 for Trends in Canadian Suicide Mortality by Age and Sex From 2000 to 2024: Tendances en matière de mortalité attribuable au suicide au Canada selon l’âge et le sexe, de 2000 à 2024 by Sara Zulyniak, Gina Dimitropoulos, Eric C. Chan and Scott B. Patten in The Canadian Journal of Psychiatry

sj-docx-2-cpa-10.1177_07067437261480922 - Supplemental material for Trends in Canadian Suicide Mortality by Age and Sex From 2000 to 2024: Tendances en matière de mortalité attribuable au suicide au Canada selon l’âge et le sexe, de 2000 à 2024 Supplemental material, sj-docx-2-cpa-10.1177_07067437261480922 for Trends in Canadian Suicide Mortality by Age and Sex From 2000 to 2024: Tendances en matière de mortalité attribuable au suicide au Canada selon l’âge et le sexe, de 2000 à 2024 by Sara Zulyniak, Gina Dimitropoulos, Eric C. Chan and Scott B. Patten in The Canadian Journal of Psychiatry

sj-docx-3-cpa-10.1177_07067437261480922 - Supplemental material for Trends in Canadian Suicide Mortality by Age and Sex From 2000 to 2024: Tendances en matière de mortalité attribuable au suicide au Canada selon l’âge et le sexe, de 2000 à 2024 Supplemental material, sj-docx-3-cpa-10.1177_07067437261480922 for Trends in Canadian Suicide Mortality by Age and Sex From 2000 to 2024: Tendances en matière de mortalité attribuable au suicide au Canada selon l’âge et le sexe, de 2000 à 2024 by Sara Zulyniak, Gina Dimitropoulos, Eric C. Chan and Scott B. Patten in The Canadian Journal of Psychiatry

sj-docx-4-cpa-10.1177_07067437261480922 - Supplemental material for Trends in Canadian Suicide Mortality by Age and Sex From 2000 to 2024: Tendances en matière de mortalité attribuable au suicide au Canada selon l’âge et le sexe, de 2000 à 2024 Supplemental material, sj-docx-4-cpa-10.1177_07067437261480922 for Trends in Canadian Suicide Mortality by Age and Sex From 2000 to 2024: Tendances en matière de mortalité attribuable au suicide au Canada selon l’âge et le sexe, de 2000 à 2024 by Sara Zulyniak, Gina Dimitropoulos, Eric C. Chan and Scott B. Patten in The Canadian Journal of Psychiatry

sj-docx-5-cpa-10.1177_07067437261480922 - Supplemental material for Trends in Canadian Suicide Mortality by Age and Sex From 2000 to 2024: Tendances en matière de mortalité attribuable au suicide au Canada selon l’âge et le sexe, de 2000 à 2024 Supplemental material, sj-docx-5-cpa-10.1177_07067437261480922 for Trends in Canadian Suicide Mortality by Age and Sex From 2000 to 2024: Tendances en matière de mortalité attribuable au suicide au Canada selon l’âge et le sexe, de 2000 à 2024 by Sara Zulyniak, Gina Dimitropoulos, Eric C. Chan and Scott B. Patten in The Canadian Journal of Psychiatry
