# Supplementary material for: Trends in Canadian Suicide Mortality by Age and Sex From 2000 to 2024: Tendances en matière de mortalité attribuable au suicide au Canada selon l’âge et le sexe, de 2000 à 2024
Source: Can J Psychiatry. 2026 Sep 3:07067437261480922. Online ahead of print. doi: 10.1177/07067437261480922 (PMC13541989; doi:10.1177/07067437261480922)
Supplement: Supplementary file 1 [file sj-docx-1-cpa-10.1177_07067437261480922.docx]

**Appendix A**. Overall, and sex-specific joinpoint graphs

Figure A.1. Overall Canadian suicide rates and predicted joinpoint regression trends from 2000 to 2024 in all ages, both sexes.

Figure A.2. Canadian suicide rates and predicted joinpoint regression trends in males of all ages from 2000 to 2024.

Figure A.3. Canadian suicide rates and predicted joinpoint regression trends in females of all ages from 2000 to 2024.
