# Supplementary material for: Trends in Canadian Suicide Mortality by Age and Sex From 2000 to 2024: Tendances en matière de mortalité attribuable au suicide au Canada selon l’âge et le sexe, de 2000 à 2024
Source: Can J Psychiatry. 2026 Sep 3:07067437261480922. Online ahead of print. doi: 10.1177/07067437261480922 (PMC13541989; doi:10.1177/07067437261480922)
Supplement: Supplementary file 2 [file sj-docx-2-cpa-10.1177_07067437261480922.docx]

**Appendix B.** Additional joinpoint graphs for other age/sex groups

Figure B.1. Observed suicide rates (per 100,000) and predicted joinpoint regression trends for 35- to 39-year-old males in Canada from 2000 to 2024.

Figure B.2. Observed suicide rates (per 100,000) and predicted joinpoint regression trends for 35- to 39-year-old females in Canada from 2000 to 2024.

Figure B.3. Observed suicide rates (per 100,000) and predicted joinpoint regression trends for 40- to 44-year-old males in Canada from 2000 to 2024.

Figure B.4. Observed suicide rates (per 100,000) and predicted joinpoint regression trends for 40- to 44-year-old females in Canada from 2000 to 2024.

Figure B.5. Observed suicide rates (per 100,000) and predicted joinpoint regression trends for 45- to 49-year-old males in Canada from 2000 to 2024.

Figure B.6. Observed suicide rates (per 100,000) and predicted joinpoint regression trends for 45- to 49-year-old females in Canada from 2000 to 2024.

Figure B.7. Observed suicide rates (per 100,000) and predicted joinpoint regression trends for 50- to 54-year-old males in Canada from 2000 to 2024.

Figure B.8. Observed suicide rates (per 100,000) and predicted joinpoint regression trends for 50- to 54-year-old females in Canada from 2000 to 2024.

Figure B.9. Observed suicide rates (per 100,000) and predicted joinpoint regression trends for 55- to 59-year-old males in Canada from 2000 to 2024.

Figure B.10. Observed suicide rates (per 100,000) and predicted joinpoint regression trends for 55- to 59-year-old females in Canada from 2000 to 2024.

Figure B.11. Observed suicide rates (per 100,000) and predicted joinpoint regression trends for 60- to 64-year-old males in Canada from 2000 to 2024.

Figure B.12. Observed suicide rates (per 100,000) and predicted joinpoint regression trends for 60- to 64-year-old females in Canada from 2000 to 2024.

Figure B.13. Observed suicide rates (per 100,000) and predicted joinpoint regression trends for 65- to 69-year-old males in Canada from 2000 to 2024.

Figure B.14. Observed suicide rates (per 100,000) and predicted joinpoint regression trends for 65- to 69-year-old females in Canada from 2000 to 2024.

Figure B.15. Observed suicide rates (per 100,000) and predicted joinpoint regression trends for 70- to 74-year-old males in Canada from 2000 to 2024.

Figure B.16. Observed suicide rates (per 100,000) and predicted joinpoint regression trends for 70- to 74-year-old females in Canada from 2000 to 2024.

Figure B.17. Observed suicide rates (per 100,000) and predicted joinpoint regression trends for 75- to 79-year-old males in Canada from 2000 to 2024.

Figure B.18. Observed suicide rates (per 100,000) and predicted joinpoint regression trends for 75- to 79-year-old females in Canada from 2000 to 2024.

Figure B.19. Observed suicide rates (per 100,000) and predicted joinpoint regression trends for 80- to 84-year-old males in Canada from 2000 to 2024.

Figure B.20. Observed suicide rates (per 100,000) and predicted joinpoint regression trends for 80- to 84-year-old females in Canada from 2000 to 2024.

Figure B.21. Observed suicide rates (per 100,000) and predicted joinpoint regression trends for 85+ year-old males in Canada from 2000 to 2024.

Figure B.22. Observed suicide rates (per 100,000) and predicted joinpoint regression trends for 85+ year-old females in Canada from 2000 to 2024.
