# Supplementary material for: Trends in Canadian Suicide Mortality by Age and Sex From 2000 to 2024: Tendances en matière de mortalité attribuable au suicide au Canada selon l’âge et le sexe, de 2000 à 2024
Source: Can J Psychiatry. 2026 Sep 3:07067437261480922. Online ahead of print. doi: 10.1177/07067437261480922 (PMC13541989; doi:10.1177/07067437261480922)
Supplement: Supplementary file 3 [file sj-docx-3-cpa-10.1177_07067437261480922.docx]

**Appendix C**. Suicide metrics of Canadian males

Table C.1. Suicide counts and rates for Canadian males of each age group.

| Age group | Year | Deaths (count) | Rate (per 100,000) | SE (per 100,000) |
| --- | --- | --- | --- | --- |
| Males | | | | |
| 10-14 | 2000 | 29 | 2.75 | 0.51 |
| 10-14 | 2001 | 15 | 1.41 | 0.36 |
| 10-14 | 2002 | 18 | 1.66 | 0.39 |
| 10-14 | 2003 | 19 | 1.73 | 0.40 |
| 10-14 | 2004 | 17 | 1.55 | 0.38 |
| 10-14 | 2005 | 18 | 1.66 | 0.39 |
| 10-14 | 2006 | 14 | 1.31 | 0.35 |
| 10-14 | 2007 | 18 | 1.70 | 0.40 |
| 10-14 | 2008 | 16 | 1.54 | 0.38 |
| 10-14 | 2009 | 13 | 1.27 | 0.35 |
| 10-14 | 2010 | 20 | 1.99 | 0.44 |
| 10-14 | 2011 | 15 | 1.52 | 0.39 |
| 10-14 | 2012 | 17 | 1.74 | 0.42 |
| 10-14 | 2013 | 15 | 1.55 | 0.40 |
| 10-14 | 2014 | 12 | 1.24 | 0.36 |
| 10-14 | 2015 | 14 | 1.44 | 0.39 |
| 10-14 | 2016 | 17 | 1.74 | 0.42 |
| 10-14 | 2017 | 11 | 1.10 | 0.33 |
| 10-14 | 2018 | 21 | 2.05 | 0.45 |
| 10-14 | 2019 | 16 | 1.52 | 0.38 |
| 10-14 | 2020 | 18 | 1.67 | 0.39 |
| 10-14 | 2021 | 10 | 0.92 | 0.29 |
| 10-14 | 2022 | 12 | 1.09 | 0.31 |
| 10-14 | 2023 | 17 | 1.51 | 0.37 |
| 10-14 | 2024 | 14 | 1.22 | 0.33 |
| 15-19 | 2000 | 174 | 16.15 | 1.22 |
| 15-19 | 2001 | 157 | 14.43 | 1.15 |
| 15-19 | 2002 | 165 | 15.07 | 1.17 |
| 15-19 | 2003 | 161 | 14.70 | 1.16 |
| 15-19 | 2004 | 161 | 14.59 | 1.15 |
| 15-19 | 2005 | 147 | 13.13 | 1.08 |
| 15-19 | 2006 | 112 | 9.86 | 0.93 |
| 15-19 | 2007 | 132 | 11.56 | 1.01 |
| 15-19 | 2008 | 142 | 12.36 | 1.04 |
| 15-19 | 2009 | 146 | 12.68 | 1.05 |
| 15-19 | 2010 | 146 | 12.70 | 1.05 |
| 15-19 | 2011 | 143 | 12.48 | 1.04 |
| 15-19 | 2012 | 162 | 14.30 | 1.12 |
| 15-19 | 2013 | 102 | 9.16 | 0.91 |
| 15-19 | 2014 | 153 | 13.95 | 1.13 |
| 15-19 | 2015 | 142 | 13.10 | 1.10 |
| 15-19 | 2016 | 130 | 12.06 | 1.06 |
| 15-19 | 2017 | 153 | 14.30 | 1.16 |
| 15-19 | 2018 | 166 | 15.52 | 1.20 |
| 15-19 | 2019 | 141 | 13.16 | 1.11 |
| 15-19 | 2020 | 118 | 11.04 | 1.02 |
| 15-19 | 2021 | 125 | 11.80 | 1.06 |
| 15-19 | 2022 | 131 | 11.88 | 1.04 |
| 15-19 | 2023 | 98 | 8.52 | 0.86 |
| 15-19 | 2024 | 89 | 7.44 | 0.79 |
| 20-24 | 2000 | 261 | 24.65 | 1.53 |
| 20-24 | 2001 | 246 | 22.80 | 1.45 |
| 20-24 | 2002 | 227 | 20.73 | 1.38 |
| 20-24 | 2003 | 251 | 22.55 | 1.42 |
| 20-24 | 2004 | 219 | 19.38 | 1.31 |
| 20-24 | 2005 | 231 | 20.21 | 1.33 |
| 20-24 | 2006 | 223 | 19.34 | 1.30 |
| 20-24 | 2007 | 231 | 19.99 | 1.31 |
| 20-24 | 2008 | 203 | 17.58 | 1.23 |
| 20-24 | 2009 | 206 | 17.71 | 1.23 |
| 20-24 | 2010 | 227 | 19.26 | 1.28 |
| 20-24 | 2011 | 238 | 19.95 | 1.29 |
| 20-24 | 2012 | 226 | 18.52 | 1.23 |
| 20-24 | 2013 | 196 | 15.78 | 1.13 |
| 20-24 | 2014 | 194 | 15.48 | 1.11 |
| 20-24 | 2015 | 233 | 18.69 | 1.22 |
| 20-24 | 2016 | 249 | 19.99 | 1.27 |
| 20-24 | 2017 | 251 | 19.93 | 1.26 |
| 20-24 | 2018 | 289 | 22.56 | 1.33 |
| 20-24 | 2019 | 267 | 20.56 | 1.26 |
| 20-24 | 2020 | 235 | 18.26 | 1.19 |
| 20-24 | 2021 | 223 | 17.87 | 1.20 |
| 20-24 | 2022 | 185 | 14.46 | 1.06 |
| 20-24 | 2023 | 181 | 13.40 | 1.00 |
| 20-24 | 2024 | 166 | 11.52 | 0.89 |
| 25-29 | 2000 | 219 | 20.84 | 1.41 |
| 25-29 | 2001 | 246 | 23.37 | 1.49 |
| 25-29 | 2002 | 220 | 20.80 | 1.40 |
| 25-29 | 2003 | 199 | 18.73 | 1.33 |
| 25-29 | 2004 | 219 | 20.40 | 1.38 |
| 25-29 | 2005 | 198 | 18.25 | 1.30 |
| 25-29 | 2006 | 179 | 16.28 | 1.22 |
| 25-29 | 2007 | 229 | 20.47 | 1.35 |
| 25-29 | 2008 | 194 | 16.97 | 1.22 |
| 25-29 | 2009 | 205 | 17.55 | 1.23 |
| 25-29 | 2010 | 194 | 16.37 | 1.18 |
| 25-29 | 2011 | 204 | 17.17 | 1.20 |
| 25-29 | 2012 | 219 | 18.27 | 1.23 |
| 25-29 | 2013 | 213 | 17.63 | 1.21 |
| 25-29 | 2014 | 230 | 18.77 | 1.24 |
| 25-29 | 2015 | 268 | 21.60 | 1.32 |
| 25-29 | 2016 | 241 | 19.04 | 1.23 |
| 25-29 | 2017 | 286 | 22.11 | 1.31 |
| 25-29 | 2018 | 266 | 19.94 | 1.22 |
| 25-29 | 2019 | 299 | 21.84 | 1.26 |
| 25-29 | 2020 | 282 | 20.35 | 1.21 |
| 25-29 | 2021 | 256 | 18.44 | 1.15 |
| 25-29 | 2022 | 226 | 15.82 | 1.05 |
| 25-29 | 2023 | 225 | 14.89 | 0.99 |
| 25-29 | 2024 | 209 | 13.22 | 0.91 |
| 30-34 | 2000 | 267 | 23.50 | 1.44 |
| 30-34 | 2001 | 262 | 23.17 | 1.43 |
| 30-34 | 2002 | 254 | 22.53 | 1.41 |
| 30-34 | 2003 | 232 | 20.72 | 1.36 |
| 30-34 | 2004 | 261 | 23.55 | 1.46 |
| 30-34 | 2005 | 220 | 20.01 | 1.35 |
| 30-34 | 2006 | 187 | 17.12 | 1.25 |
| 30-34 | 2007 | 182 | 16.64 | 1.23 |
| 30-34 | 2008 | 202 | 18.30 | 1.29 |
| 30-34 | 2009 | 235 | 20.97 | 1.37 |
| 30-34 | 2010 | 217 | 19.06 | 1.29 |
| 30-34 | 2011 | 235 | 20.26 | 1.32 |
| 30-34 | 2012 | 208 | 17.64 | 1.22 |
| 30-34 | 2013 | 226 | 18.83 | 1.25 |
| 30-34 | 2014 | 238 | 19.51 | 1.26 |
| 30-34 | 2015 | 237 | 19.30 | 1.25 |
| 30-34 | 2016 | 246 | 19.73 | 1.26 |
| 30-34 | 2017 | 266 | 21.07 | 1.29 |
| 30-34 | 2018 | 254 | 19.77 | 1.24 |
| 30-34 | 2019 | 295 | 22.42 | 1.31 |
| 30-34 | 2020 | 280 | 20.75 | 1.24 |
| 30-34 | 2021 | 264 | 19.20 | 1.18 |
| 30-34 | 2022 | 292 | 20.42 | 1.19 |
| 30-34 | 2023 | 251 | 16.41 | 1.04 |
| 30-34 | 2024 | 251 | 15.52 | 0.98 |
| 35-39 | 2000 | 347 | 25.62 | 1.37 |
| 35-39 | 2001 | 356 | 26.75 | 1.42 |
| 35-39 | 2002 | 320 | 24.60 | 1.38 |
| 35-39 | 2003 | 346 | 27.56 | 1.48 |
| 35-39 | 2004 | 284 | 23.39 | 1.39 |
| 35-39 | 2005 | 299 | 25.24 | 1.46 |
| 35-39 | 2006 | 261 | 22.25 | 1.38 |
| 35-39 | 2007 | 258 | 22.18 | 1.38 |
| 35-39 | 2008 | 240 | 20.73 | 1.34 |
| 35-39 | 2009 | 254 | 22.11 | 1.39 |
| 35-39 | 2010 | 267 | 23.38 | 1.43 |
| 35-39 | 2011 | 236 | 20.82 | 1.35 |
| 35-39 | 2012 | 228 | 20.00 | 1.32 |
| 35-39 | 2013 | 222 | 19.30 | 1.30 |
| 35-39 | 2014 | 248 | 21.32 | 1.35 |
| 35-39 | 2015 | 225 | 19.20 | 1.28 |
| 35-39 | 2016 | 256 | 21.50 | 1.34 |
| 35-39 | 2017 | 271 | 22.26 | 1.35 |
| 35-39 | 2018 | 269 | 21.47 | 1.31 |
| 35-39 | 2019 | 286 | 22.17 | 1.31 |
| 35-39 | 2020 | 273 | 20.74 | 1.26 |
| 35-39 | 2021 | 281 | 21.10 | 1.26 |
| 35-39 | 2022 | 280 | 20.56 | 1.23 |
| 35-39 | 2023 | 275 | 19.32 | 1.16 |
| 35-39 | 2024 | 237 | 15.79 | 1.03 |
| 40-44 | 2000 | 393 | 30.24 | 1.53 |
| 40-44 | 2001 | 360 | 27.07 | 1.43 |
| 40-44 | 2002 | 346 | 25.62 | 1.38 |
| 40-44 | 2003 | 358 | 26.14 | 1.38 |
| 40-44 | 2004 | 320 | 23.08 | 1.29 |
| 40-44 | 2005 | 374 | 26.89 | 1.39 |
| 40-44 | 2006 | 332 | 24.23 | 1.33 |
| 40-44 | 2007 | 299 | 22.44 | 1.30 |
| 40-44 | 2008 | 356 | 27.73 | 1.47 |
| 40-44 | 2009 | 340 | 27.42 | 1.49 |
| 40-44 | 2010 | 272 | 22.54 | 1.37 |
| 40-44 | 2011 | 280 | 23.43 | 1.40 |
| 40-44 | 2012 | 296 | 24.86 | 1.44 |
| 40-44 | 2013 | 273 | 23.03 | 1.39 |
| 40-44 | 2014 | 279 | 23.74 | 1.42 |
| 40-44 | 2015 | 276 | 23.69 | 1.43 |
| 40-44 | 2016 | 297 | 25.65 | 1.49 |
| 40-44 | 2017 | 260 | 22.36 | 1.39 |
| 40-44 | 2018 | 272 | 23.07 | 1.40 |
| 40-44 | 2019 | 282 | 23.44 | 1.40 |
| 40-44 | 2020 | 271 | 22.10 | 1.34 |
| 40-44 | 2021 | 251 | 20.02 | 1.26 |
| 40-44 | 2022 | 282 | 21.89 | 1.30 |
| 40-44 | 2023 | 254 | 18.88 | 1.18 |
| 40-44 | 2024 | 274 | 19.42 | 1.17 |
| 45-49 | 2000 | 290 | 25.22 | 1.48 |
| 45-49 | 2001 | 347 | 29.37 | 1.58 |
| 45-49 | 2002 | 362 | 29.65 | 1.56 |
| 45-49 | 2003 | 332 | 26.41 | 1.45 |
| 45-49 | 2004 | 328 | 25.53 | 1.41 |
| 45-49 | 2005 | 380 | 29.01 | 1.49 |
| 45-49 | 2006 | 352 | 26.30 | 1.40 |
| 45-49 | 2007 | 369 | 27.13 | 1.41 |
| 45-49 | 2008 | 338 | 24.42 | 1.33 |
| 45-49 | 2009 | 378 | 26.98 | 1.39 |
| 45-49 | 2010 | 406 | 29.02 | 1.44 |
| 45-49 | 2011 | 334 | 24.38 | 1.33 |
| 45-49 | 2012 | 338 | 25.30 | 1.38 |
| 45-49 | 2013 | 369 | 28.55 | 1.49 |
| 45-49 | 2014 | 359 | 28.69 | 1.51 |
| 45-49 | 2015 | 356 | 29.20 | 1.55 |
| 45-49 | 2016 | 319 | 26.40 | 1.48 |
| 45-49 | 2017 | 313 | 26.07 | 1.47 |
| 45-49 | 2018 | 334 | 27.95 | 1.53 |
| 45-49 | 2019 | 311 | 26.14 | 1.48 |
| 45-49 | 2020 | 272 | 22.92 | 1.39 |
| 45-49 | 2021 | 249 | 21.07 | 1.33 |
| 45-49 | 2022 | 283 | 23.71 | 1.41 |
| 45-49 | 2023 | 247 | 20.19 | 1.28 |
| 45-49 | 2024 | 237 | 18.70 | 1.21 |
| 50-54 | 2000 | 240 | 23.55 | 1.52 |
| 50-54 | 2001 | 306 | 29.04 | 1.66 |
| 50-54 | 2002 | 292 | 27.44 | 1.61 |
| 50-54 | 2003 | 286 | 26.46 | 1.56 |
| 50-54 | 2004 | 287 | 25.85 | 1.53 |
| 50-54 | 2005 | 296 | 25.81 | 1.50 |
| 50-54 | 2006 | 272 | 23.01 | 1.40 |
| 50-54 | 2007 | 314 | 25.68 | 1.45 |
| 50-54 | 2008 | 311 | 24.65 | 1.40 |
| 50-54 | 2009 | 367 | 28.39 | 1.48 |
| 50-54 | 2010 | 355 | 26.89 | 1.43 |
| 50-54 | 2011 | 338 | 25.03 | 1.36 |
| 50-54 | 2012 | 362 | 26.48 | 1.39 |
| 50-54 | 2013 | 381 | 27.55 | 1.41 |
| 50-54 | 2014 | 391 | 28.02 | 1.42 |
| 50-54 | 2015 | 379 | 27.22 | 1.40 |
| 50-54 | 2016 | 363 | 26.56 | 1.39 |
| 50-54 | 2017 | 362 | 27.24 | 1.43 |
| 50-54 | 2018 | 359 | 27.98 | 1.48 |
| 50-54 | 2019 | 325 | 26.17 | 1.45 |
| 50-54 | 2020 | 257 | 21.22 | 1.32 |
| 50-54 | 2021 | 249 | 20.73 | 1.31 |
| 50-54 | 2022 | 258 | 21.52 | 1.34 |
| 50-54 | 2023 | 275 | 22.89 | 1.38 |
| 50-54 | 2024 | 261 | 21.59 | 1.34 |
| 55-59 | 2000 | 167 | 21.62 | 1.67 |
| 55-59 | 2001 | 195 | 24.09 | 1.72 |
| 55-59 | 2002 | 208 | 23.93 | 1.66 |
| 55-59 | 2003 | 231 | 25.20 | 1.66 |
| 55-59 | 2004 | 206 | 21.52 | 1.50 |
| 55-59 | 2005 | 222 | 22.26 | 1.49 |
| 55-59 | 2006 | 253 | 24.50 | 1.54 |
| 55-59 | 2007 | 227 | 21.71 | 1.44 |
| 55-59 | 2008 | 236 | 22.11 | 1.44 |
| 55-59 | 2009 | 291 | 26.47 | 1.55 |
| 55-59 | 2010 | 276 | 24.26 | 1.46 |
| 55-59 | 2011 | 301 | 25.65 | 1.48 |
| 55-59 | 2012 | 333 | 27.49 | 1.51 |
| 55-59 | 2013 | 320 | 25.62 | 1.43 |
| 55-59 | 2014 | 350 | 27.41 | 1.46 |
| 55-59 | 2015 | 365 | 28.08 | 1.47 |
| 55-59 | 2016 | 368 | 27.79 | 1.45 |
| 55-59 | 2017 | 352 | 26.25 | 1.40 |
| 55-59 | 2018 | 378 | 27.82 | 1.43 |
| 55-59 | 2019 | 378 | 27.55 | 1.42 |
| 55-59 | 2020 | 366 | 26.74 | 1.40 |
| 55-59 | 2021 | 268 | 20.01 | 1.22 |
| 55-59 | 2022 | 282 | 21.64 | 1.29 |
| 55-59 | 2023 | 309 | 24.50 | 1.39 |
| 55-59 | 2024 | 277 | 22.55 | 1.35 |
| 60-64 | 2000 | 123 | 20.05 | 1.81 |
| 60-64 | 2001 | 117 | 18.54 | 1.71 |
| 60-64 | 2002 | 128 | 19.52 | 1.73 |
| 60-64 | 2003 | 155 | 22.55 | 1.81 |
| 60-64 | 2004 | 126 | 17.50 | 1.56 |
| 60-64 | 2005 | 130 | 17.27 | 1.51 |
| 60-64 | 2006 | 176 | 22.33 | 1.68 |
| 60-64 | 2007 | 153 | 18.12 | 1.46 |
| 60-64 | 2008 | 149 | 16.72 | 1.37 |
| 60-64 | 2009 | 182 | 19.50 | 1.45 |
| 60-64 | 2010 | 202 | 20.66 | 1.45 |
| 60-64 | 2011 | 189 | 18.65 | 1.36 |
| 60-64 | 2012 | 215 | 21.01 | 1.43 |
| 60-64 | 2013 | 240 | 23.05 | 1.49 |
| 60-64 | 2014 | 226 | 21.14 | 1.41 |
| 60-64 | 2015 | 289 | 26.21 | 1.54 |
| 60-64 | 2016 | 253 | 22.28 | 1.40 |
| 60-64 | 2017 | 265 | 22.60 | 1.39 |
| 60-64 | 2018 | 278 | 22.99 | 1.38 |
| 60-64 | 2019 | 317 | 25.58 | 1.44 |
| 60-64 | 2020 | 258 | 20.40 | 1.27 |
| 60-64 | 2021 | 257 | 19.94 | 1.24 |
| 60-64 | 2022 | 307 | 23.52 | 1.34 |
| 60-64 | 2023 | 247 | 18.67 | 1.19 |
| 60-64 | 2024 | 275 | 20.55 | 1.24 |
| 65-69 | 2000 | 93 | 16.91 | 1.75 |
| 65-69 | 2001 | 72 | 13.09 | 1.54 |
| 65-69 | 2002 | 102 | 18.59 | 1.84 |
| 65-69 | 2003 | 110 | 19.91 | 1.90 |
| 65-69 | 2004 | 82 | 14.57 | 1.61 |
| 65-69 | 2005 | 104 | 18.11 | 1.78 |
| 65-69 | 2006 | 107 | 17.99 | 1.74 |
| 65-69 | 2007 | 89 | 14.36 | 1.52 |
| 65-69 | 2008 | 114 | 17.57 | 1.65 |
| 65-69 | 2009 | 113 | 16.64 | 1.57 |
| 65-69 | 2010 | 118 | 16.63 | 1.53 |
| 65-69 | 2011 | 122 | 16.38 | 1.48 |
| 65-69 | 2012 | 135 | 16.85 | 1.45 |
| 65-69 | 2013 | 157 | 18.49 | 1.48 |
| 65-69 | 2014 | 159 | 17.90 | 1.42 |
| 65-69 | 2015 | 183 | 19.76 | 1.46 |
| 65-69 | 2016 | 205 | 21.41 | 1.50 |
| 65-69 | 2017 | 180 | 18.57 | 1.38 |
| 65-69 | 2018 | 180 | 18.24 | 1.36 |
| 65-69 | 2019 | 194 | 19.11 | 1.37 |
| 65-69 | 2020 | 184 | 17.55 | 1.29 |
| 65-69 | 2021 | 184 | 17.04 | 1.26 |
| 65-69 | 2022 | 207 | 18.52 | 1.29 |
| 65-69 | 2023 | 174 | 15.07 | 1.14 |
| 65-69 | 2024 | 191 | 16.10 | 1.16 |
| 70-74 | 2000 | 78 | 17.00 | 1.92 |
| 70-74 | 2001 | 100 | 21.30 | 2.13 |
| 70-74 | 2002 | 101 | 21.08 | 2.10 |
| 70-74 | 2003 | 87 | 17.96 | 1.93 |
| 70-74 | 2004 | 83 | 17.03 | 1.87 |
| 70-74 | 2005 | 83 | 17.00 | 1.87 |
| 70-74 | 2006 | 69 | 14.08 | 1.70 |
| 70-74 | 2007 | 76 | 15.48 | 1.78 |
| 70-74 | 2008 | 90 | 18.09 | 1.91 |
| 70-74 | 2009 | 106 | 20.78 | 2.02 |
| 70-74 | 2010 | 98 | 18.70 | 1.89 |
| 70-74 | 2011 | 112 | 20.64 | 1.95 |
| 70-74 | 2012 | 109 | 19.32 | 1.85 |
| 70-74 | 2013 | 101 | 17.06 | 1.70 |
| 70-74 | 2014 | 109 | 17.55 | 1.68 |
| 70-74 | 2015 | 117 | 18.01 | 1.67 |
| 70-74 | 2016 | 94 | 13.77 | 1.42 |
| 70-74 | 2017 | 129 | 17.52 | 1.54 |
| 70-74 | 2018 | 134 | 17.18 | 1.48 |
| 70-74 | 2019 | 151 | 18.45 | 1.50 |
| 70-74 | 2020 | 132 | 15.43 | 1.34 |
| 70-74 | 2021 | 118 | 13.33 | 1.23 |
| 70-74 | 2022 | 148 | 16.53 | 1.36 |
| 70-74 | 2023 | 128 | 14.02 | 1.24 |
| 70-74 | 2024 | 154 | 16.37 | 1.32 |
| 75-79 | 2000 | 79 | 23.62 | 2.66 |
| 75-79 | 2001 | 71 | 20.69 | 2.46 |
| 75-79 | 2002 | 73 | 20.83 | 2.44 |
| 75-79 | 2003 | 69 | 19.16 | 2.31 |
| 75-79 | 2004 | 71 | 19.29 | 2.29 |
| 75-79 | 2005 | 86 | 22.80 | 2.46 |
| 75-79 | 2006 | 92 | 23.76 | 2.48 |
| 75-79 | 2007 | 83 | 20.94 | 2.30 |
| 75-79 | 2008 | 90 | 22.33 | 2.35 |
| 75-79 | 2009 | 69 | 16.93 | 2.04 |
| 75-79 | 2010 | 80 | 19.45 | 2.17 |
| 75-79 | 2011 | 84 | 20.21 | 2.20 |
| 75-79 | 2012 | 69 | 16.42 | 1.98 |
| 75-79 | 2013 | 84 | 19.59 | 2.14 |
| 75-79 | 2014 | 95 | 21.56 | 2.21 |
| 75-79 | 2015 | 91 | 20.12 | 2.11 |
| 75-79 | 2016 | 104 | 22.22 | 2.18 |
| 75-79 | 2017 | 89 | 18.20 | 1.93 |
| 75-79 | 2018 | 116 | 22.52 | 2.09 |
| 75-79 | 2019 | 90 | 16.59 | 1.75 |
| 75-79 | 2020 | 107 | 18.80 | 1.82 |
| 75-79 | 2021 | 81 | 13.55 | 1.50 |
| 75-79 | 2022 | 103 | 15.96 | 1.57 |
| 75-79 | 2023 | 80 | 11.67 | 1.30 |
| 75-79 | 2024 | 105 | 14.57 | 1.42 |
| 80-84 | 2000 | 38 | 20.60 | 3.34 |
| 80-84 | 2001 | 27 | 13.78 | 2.65 |
| 80-84 | 2002 | 46 | 21.97 | 3.24 |
| 80-84 | 2003 | 44 | 19.83 | 2.99 |
| 80-84 | 2004 | 48 | 20.52 | 2.96 |
| 80-84 | 2005 | 47 | 19.33 | 2.82 |
| 80-84 | 2006 | 47 | 18.67 | 2.72 |
| 80-84 | 2007 | 61 | 23.73 | 3.04 |
| 80-84 | 2008 | 59 | 22.30 | 2.90 |
| 80-84 | 2009 | 54 | 19.89 | 2.71 |
| 80-84 | 2010 | 71 | 25.30 | 3.00 |
| 80-84 | 2011 | 63 | 21.69 | 2.73 |
| 80-84 | 2012 | 62 | 20.67 | 2.63 |
| 80-84 | 2013 | 74 | 24.09 | 2.80 |
| 80-84 | 2014 | 79 | 25.23 | 2.84 |
| 80-84 | 2015 | 86 | 27.08 | 2.92 |
| 80-84 | 2016 | 76 | 23.50 | 2.69 |
| 80-84 | 2017 | 71 | 21.66 | 2.57 |
| 80-84 | 2018 | 68 | 20.31 | 2.46 |
| 80-84 | 2019 | 70 | 20.25 | 2.42 |
| 80-84 | 2020 | 62 | 17.37 | 2.21 |
| 80-84 | 2021 | 61 | 16.43 | 2.10 |
| 80-84 | 2022 | 77 | 19.82 | 2.26 |
| 80-84 | 2023 | 48 | 11.70 | 1.69 |
| 80-84 | 2024 | 79 | 18.24 | 2.05 |
| 85+ | 2000 | 30 | 24.88 | 4.54 |
| 85+ | 2001 | 34 | 27.18 | 4.66 |
| 85+ | 2002 | 45 | 34.79 | 5.19 |
| 85+ | 2003 | 38 | 28.37 | 4.60 |
| 85+ | 2004 | 35 | 25.23 | 4.26 |
| 85+ | 2005 | 31 | 20.99 | 3.77 |
| 85+ | 2006 | 53 | 33.16 | 4.55 |
| 85+ | 2007 | 39 | 22.89 | 3.66 |
| 85+ | 2008 | 46 | 25.56 | 3.77 |
| 85+ | 2009 | 59 | 31.10 | 4.05 |
| 85+ | 2010 | 51 | 25.53 | 3.57 |
| 85+ | 2011 | 47 | 22.44 | 3.27 |
| 85+ | 2012 | 51 | 23.06 | 3.23 |
| 85+ | 2013 | 73 | 31.37 | 3.67 |
| 85+ | 2014 | 66 | 27.02 | 3.33 |
| 85+ | 2015 | 69 | 27.02 | 3.25 |
| 85+ | 2016 | 65 | 24.14 | 2.99 |
| 85+ | 2017 | 79 | 28.19 | 3.17 |
| 85+ | 2018 | 70 | 24.18 | 2.89 |
| 85+ | 2019 | 85 | 28.51 | 3.09 |
| 85+ | 2020 | 84 | 27.54 | 3.00 |
| 85+ | 2021 | 61 | 19.40 | 2.48 |
| 85+ | 2022 | 69 | 21.40 | 2.58 |
| 85+ | 2023 | 60 | 18.01 | 2.33 |
| 85+ | 2024 | 61 | 17.64 | 2.26 |
