# Supplementary material for: Trends in Canadian Suicide Mortality by Age and Sex From 2000 to 2024: Tendances en matière de mortalité attribuable au suicide au Canada selon l’âge et le sexe, de 2000 à 2024
Source: Can J Psychiatry. 2026 Sep 3:07067437261480922. Online ahead of print. doi: 10.1177/07067437261480922 (PMC13541989; doi:10.1177/07067437261480922)
Supplement: Supplementary file 4 [file sj-docx-4-cpa-10.1177_07067437261480922.docx]

**Appendix D**. Suicide metrics of Canadian females

Table D.1. Suicide counts and rates for Canadian females of each age group.

| Age group | Year | Deaths (count) | Rate (per 100,000) | SE (per 100,000) |
| --- | --- | --- | --- | --- |
| Females | | | | |
| 10-14 | 2000 | 18 | 1.80 | 0.42 |
| 10-14 | 2001 | 14 | 1.38 | 0.37 |
| 10-14 | 2002 | 18 | 1.75 | 0.41 |
| 10-14 | 2003 | 8 | 0.77 | 0.27 |
| 10-14 | 2004 | 11 | 1.05 | 0.32 |
| 10-14 | 2005 | 25 | 2.41 | 0.48 |
| 10-14 | 2006 | 18 | 1.76 | 0.41 |
| 10-14 | 2007 | 15 | 1.49 | 0.38 |
| 10-14 | 2008 | 9 | 0.91 | 0.30 |
| 10-14 | 2009 | 12 | 1.24 | 0.36 |
| 10-14 | 2010 | 13 | 1.36 | 0.38 |
| 10-14 | 2011 | 17 | 1.83 | 0.44 |
| 10-14 | 2012 | 17 | 1.85 | 0.45 |
| 10-14 | 2013 | 20 | 2.18 | 0.49 |
| 10-14 | 2014 | 21 | 2.27 | 0.50 |
| 10-14 | 2015 | 21 | 2.27 | 0.49 |
| 10-14 | 2016 | 33 | 3.51 | 0.61 |
| 10-14 | 2017 | 26 | 2.72 | 0.53 |
| 10-14 | 2018 | 28 | 2.87 | 0.54 |
| 10-14 | 2019 | 20 | 2.01 | 0.45 |
| 10-14 | 2020 | 20 | 1.97 | 0.44 |
| 10-14 | 2021 | 23 | 2.24 | 0.47 |
| 10-14 | 2022 | 22 | 2.11 | 0.45 |
| 10-14 | 2023 | 25 | 2.35 | 0.47 |
| 10-14 | 2024 | 24 | 2.21 | 0.45 |
| 15-19 | 2000 | 52 | 5.11 | 0.71 |
| 15-19 | 2001 | 51 | 4.96 | 0.69 |
| 15-19 | 2002 | 56 | 5.42 | 0.72 |
| 15-19 | 2003 | 56 | 5.41 | 0.72 |
| 15-19 | 2004 | 49 | 4.71 | 0.67 |
| 15-19 | 2005 | 66 | 6.25 | 0.77 |
| 15-19 | 2006 | 44 | 4.09 | 0.62 |
| 15-19 | 2007 | 54 | 4.96 | 0.67 |
| 15-19 | 2008 | 68 | 6.17 | 0.75 |
| 15-19 | 2009 | 58 | 5.25 | 0.69 |
| 15-19 | 2010 | 53 | 4.82 | 0.66 |
| 15-19 | 2011 | 62 | 5.68 | 0.72 |
| 15-19 | 2012 | 68 | 6.38 | 0.77 |
| 15-19 | 2013 | 66 | 6.32 | 0.78 |
| 15-19 | 2014 | 59 | 5.74 | 0.75 |
| 15-19 | 2015 | 63 | 6.20 | 0.78 |
| 15-19 | 2016 | 68 | 6.72 | 0.81 |
| 15-19 | 2017 | 79 | 7.81 | 0.88 |
| 15-19 | 2018 | 64 | 6.31 | 0.79 |
| 15-19 | 2019 | 82 | 8.05 | 0.89 |
| 15-19 | 2020 | 67 | 6.61 | 0.81 |
| 15-19 | 2021 | 73 | 7.30 | 0.85 |
| 15-19 | 2022 | 72 | 6.93 | 0.82 |
| 15-19 | 2023 | 58 | 5.37 | 0.71 |
| 15-19 | 2024 | 52 | 4.65 | 0.64 |
| 20-24 | 2000 | 61 | 6.04 | 0.77 |
| 20-24 | 2001 | 54 | 5.24 | 0.71 |
| 20-24 | 2002 | 56 | 5.35 | 0.71 |
| 20-24 | 2003 | 58 | 5.45 | 0.72 |
| 20-24 | 2004 | 53 | 4.90 | 0.67 |
| 20-24 | 2005 | 64 | 5.86 | 0.73 |
| 20-24 | 2006 | 46 | 4.19 | 0.62 |
| 20-24 | 2007 | 61 | 5.52 | 0.71 |
| 20-24 | 2008 | 51 | 4.60 | 0.64 |
| 20-24 | 2009 | 74 | 6.59 | 0.77 |
| 20-24 | 2010 | 63 | 5.51 | 0.69 |
| 20-24 | 2011 | 77 | 6.64 | 0.76 |
| 20-24 | 2012 | 70 | 5.98 | 0.71 |
| 20-24 | 2013 | 71 | 6.04 | 0.72 |
| 20-24 | 2014 | 73 | 6.23 | 0.73 |
| 20-24 | 2015 | 89 | 7.72 | 0.82 |
| 20-24 | 2016 | 82 | 7.14 | 0.79 |
| 20-24 | 2017 | 92 | 7.94 | 0.83 |
| 20-24 | 2018 | 105 | 8.93 | 0.87 |
| 20-24 | 2019 | 88 | 7.39 | 0.79 |
| 20-24 | 2020 | 95 | 8.01 | 0.82 |
| 20-24 | 2021 | 79 | 6.83 | 0.77 |
| 20-24 | 2022 | 96 | 8.10 | 0.83 |
| 20-24 | 2023 | 73 | 5.89 | 0.69 |
| 20-24 | 2024 | 86 | 6.61 | 0.71 |
| 25-29 | 2000 | 61 | 5.96 | 0.76 |
| 25-29 | 2001 | 49 | 4.80 | 0.69 |
| 25-29 | 2002 | 49 | 4.77 | 0.68 |
| 25-29 | 2003 | 48 | 4.63 | 0.67 |
| 25-29 | 2004 | 56 | 5.33 | 0.71 |
| 25-29 | 2005 | 34 | 3.19 | 0.55 |
| 25-29 | 2006 | 63 | 5.80 | 0.73 |
| 25-29 | 2007 | 60 | 5.43 | 0.70 |
| 25-29 | 2008 | 63 | 5.58 | 0.70 |
| 25-29 | 2009 | 56 | 4.85 | 0.65 |
| 25-29 | 2010 | 78 | 6.65 | 0.75 |
| 25-29 | 2011 | 68 | 5.76 | 0.70 |
| 25-29 | 2012 | 58 | 4.90 | 0.64 |
| 25-29 | 2013 | 67 | 5.66 | 0.69 |
| 25-29 | 2014 | 92 | 7.74 | 0.81 |
| 25-29 | 2015 | 84 | 7.05 | 0.77 |
| 25-29 | 2016 | 96 | 7.98 | 0.81 |
| 25-29 | 2017 | 88 | 7.17 | 0.76 |
| 25-29 | 2018 | 87 | 6.92 | 0.74 |
| 25-29 | 2019 | 106 | 8.25 | 0.80 |
| 25-29 | 2020 | 88 | 6.80 | 0.73 |
| 25-29 | 2021 | 93 | 7.23 | 0.75 |
| 25-29 | 2022 | 97 | 7.35 | 0.75 |
| 25-29 | 2023 | 93 | 6.71 | 0.70 |
| 25-29 | 2024 | 88 | 6.07 | 0.65 |
| 30-34 | 2000 | 59 | 5.28 | 0.69 |
| 30-34 | 2001 | 66 | 5.95 | 0.73 |
| 30-34 | 2002 | 79 | 7.16 | 0.81 |
| 30-34 | 2003 | 67 | 6.12 | 0.75 |
| 30-34 | 2004 | 57 | 5.25 | 0.70 |
| 30-34 | 2005 | 63 | 5.82 | 0.73 |
| 30-34 | 2006 | 58 | 5.38 | 0.71 |
| 30-34 | 2007 | 58 | 5.33 | 0.70 |
| 30-34 | 2008 | 55 | 4.98 | 0.67 |
| 30-34 | 2009 | 64 | 5.69 | 0.71 |
| 30-34 | 2010 | 76 | 6.64 | 0.76 |
| 30-34 | 2011 | 76 | 6.51 | 0.75 |
| 30-34 | 2012 | 74 | 6.23 | 0.72 |
| 30-34 | 2013 | 68 | 5.62 | 0.68 |
| 30-34 | 2014 | 78 | 6.37 | 0.72 |
| 30-34 | 2015 | 102 | 8.30 | 0.82 |
| 30-34 | 2016 | 80 | 6.46 | 0.72 |
| 30-34 | 2017 | 81 | 6.51 | 0.72 |
| 30-34 | 2018 | 79 | 6.27 | 0.71 |
| 30-34 | 2019 | 72 | 5.61 | 0.66 |
| 30-34 | 2020 | 109 | 8.31 | 0.80 |
| 30-34 | 2021 | 92 | 6.92 | 0.72 |
| 30-34 | 2022 | 86 | 6.25 | 0.67 |
| 30-34 | 2023 | 84 | 5.78 | 0.63 |
| 30-34 | 2024 | 87 | 5.69 | 0.61 |
| 35-39 | 2000 | 87 | 6.52 | 0.70 |
| 35-39 | 2001 | 91 | 6.96 | 0.73 |
| 35-39 | 2002 | 101 | 7.91 | 0.79 |
| 35-39 | 2003 | 92 | 7.45 | 0.78 |
| 35-39 | 2004 | 109 | 9.14 | 0.88 |
| 35-39 | 2005 | 84 | 7.23 | 0.79 |
| 35-39 | 2006 | 62 | 5.39 | 0.68 |
| 35-39 | 2007 | 69 | 6.03 | 0.73 |
| 35-39 | 2008 | 76 | 6.66 | 0.76 |
| 35-39 | 2009 | 83 | 7.29 | 0.80 |
| 35-39 | 2010 | 80 | 7.02 | 0.78 |
| 35-39 | 2011 | 72 | 6.32 | 0.75 |
| 35-39 | 2012 | 72 | 6.25 | 0.74 |
| 35-39 | 2013 | 78 | 6.69 | 0.76 |
| 35-39 | 2014 | 77 | 6.51 | 0.74 |
| 35-39 | 2015 | 82 | 6.85 | 0.76 |
| 35-39 | 2016 | 83 | 6.83 | 0.75 |
| 35-39 | 2017 | 80 | 6.48 | 0.72 |
| 35-39 | 2018 | 81 | 6.43 | 0.71 |
| 35-39 | 2019 | 87 | 6.78 | 0.73 |
| 35-39 | 2020 | 89 | 6.83 | 0.72 |
| 35-39 | 2021 | 72 | 5.49 | 0.65 |
| 35-39 | 2022 | 109 | 8.15 | 0.78 |
| 35-39 | 2023 | 83 | 5.99 | 0.66 |
| 35-39 | 2024 | 93 | 6.41 | 0.67 |
| 40-44 | 2000 | 105 | 8.07 | 0.79 |
| 40-44 | 2001 | 96 | 7.24 | 0.74 |
| 40-44 | 2002 | 95 | 7.08 | 0.73 |
| 40-44 | 2003 | 106 | 7.82 | 0.76 |
| 40-44 | 2004 | 91 | 6.65 | 0.70 |
| 40-44 | 2005 | 121 | 8.84 | 0.80 |
| 40-44 | 2006 | 92 | 6.84 | 0.71 |
| 40-44 | 2007 | 111 | 8.48 | 0.81 |
| 40-44 | 2008 | 98 | 7.75 | 0.78 |
| 40-44 | 2009 | 94 | 7.67 | 0.79 |
| 40-44 | 2010 | 97 | 8.11 | 0.82 |
| 40-44 | 2011 | 90 | 7.56 | 0.80 |
| 40-44 | 2012 | 75 | 6.30 | 0.73 |
| 40-44 | 2013 | 98 | 8.25 | 0.83 |
| 40-44 | 2014 | 94 | 7.93 | 0.82 |
| 40-44 | 2015 | 98 | 8.29 | 0.84 |
| 40-44 | 2016 | 85 | 7.20 | 0.78 |
| 40-44 | 2017 | 84 | 7.08 | 0.77 |
| 40-44 | 2018 | 85 | 7.09 | 0.77 |
| 40-44 | 2019 | 87 | 7.15 | 0.77 |
| 40-44 | 2020 | 88 | 7.13 | 0.76 |
| 40-44 | 2021 | 72 | 5.75 | 0.68 |
| 40-44 | 2022 | 100 | 7.80 | 0.78 |
| 40-44 | 2023 | 80 | 6.01 | 0.67 |
| 40-44 | 2024 | 65 | 4.69 | 0.58 |
| 45-49 | 2000 | 113 | 9.70 | 0.91 |
| 45-49 | 2001 | 118 | 9.85 | 0.91 |
| 45-49 | 2002 | 90 | 7.29 | 0.77 |
| 45-49 | 2003 | 123 | 9.71 | 0.88 |
| 45-49 | 2004 | 121 | 9.38 | 0.85 |
| 45-49 | 2005 | 101 | 7.71 | 0.77 |
| 45-49 | 2006 | 113 | 8.47 | 0.80 |
| 45-49 | 2007 | 119 | 8.81 | 0.81 |
| 45-49 | 2008 | 132 | 9.63 | 0.84 |
| 45-49 | 2009 | 116 | 8.39 | 0.78 |
| 45-49 | 2010 | 96 | 6.95 | 0.71 |
| 45-49 | 2011 | 118 | 8.74 | 0.80 |
| 45-49 | 2012 | 133 | 10.06 | 0.87 |
| 45-49 | 2013 | 104 | 8.10 | 0.79 |
| 45-49 | 2014 | 120 | 9.59 | 0.88 |
| 45-49 | 2015 | 110 | 8.98 | 0.86 |
| 45-49 | 2016 | 105 | 8.60 | 0.84 |
| 45-49 | 2017 | 106 | 8.72 | 0.85 |
| 45-49 | 2018 | 103 | 8.50 | 0.84 |
| 45-49 | 2019 | 82 | 6.79 | 0.75 |
| 45-49 | 2020 | 89 | 7.38 | 0.78 |
| 45-49 | 2021 | 72 | 6.00 | 0.71 |
| 45-49 | 2022 | 82 | 6.78 | 0.75 |
| 45-49 | 2023 | 78 | 6.32 | 0.72 |
| 45-49 | 2024 | 77 | 6.06 | 0.69 |
| 50-54 | 2000 | 89 | 8.68 | 0.92 |
| 50-54 | 2001 | 91 | 8.55 | 0.90 |
| 50-54 | 2002 | 88 | 8.17 | 0.87 |
| 50-54 | 2003 | 117 | 10.65 | 0.98 |
| 50-54 | 2004 | 108 | 9.56 | 0.92 |
| 50-54 | 2005 | 116 | 9.94 | 0.92 |
| 50-54 | 2006 | 104 | 8.66 | 0.85 |
| 50-54 | 2007 | 101 | 8.18 | 0.81 |
| 50-54 | 2008 | 109 | 8.61 | 0.82 |
| 50-54 | 2009 | 113 | 8.76 | 0.82 |
| 50-54 | 2010 | 132 | 10.05 | 0.87 |
| 50-54 | 2011 | 132 | 9.84 | 0.86 |
| 50-54 | 2012 | 112 | 8.24 | 0.78 |
| 50-54 | 2013 | 119 | 8.64 | 0.79 |
| 50-54 | 2014 | 142 | 10.21 | 0.86 |
| 50-54 | 2015 | 155 | 11.15 | 0.90 |
| 50-54 | 2016 | 147 | 10.76 | 0.89 |
| 50-54 | 2017 | 135 | 10.13 | 0.87 |
| 50-54 | 2018 | 133 | 10.30 | 0.89 |
| 50-54 | 2019 | 122 | 9.71 | 0.88 |
| 50-54 | 2020 | 104 | 8.45 | 0.83 |
| 50-54 | 2021 | 90 | 7.34 | 0.77 |
| 50-54 | 2022 | 72 | 5.87 | 0.69 |
| 50-54 | 2023 | 74 | 6.02 | 0.70 |
| 50-54 | 2024 | 90 | 7.28 | 0.77 |
| 55-59 | 2000 | 50 | 6.38 | 0.90 |
| 55-59 | 2001 | 66 | 8.05 | 0.99 |
| 55-59 | 2002 | 63 | 7.15 | 0.90 |
| 55-59 | 2003 | 61 | 6.55 | 0.84 |
| 55-59 | 2004 | 69 | 7.07 | 0.85 |
| 55-59 | 2005 | 73 | 7.17 | 0.84 |
| 55-59 | 2006 | 75 | 7.11 | 0.82 |
| 55-59 | 2007 | 84 | 7.88 | 0.86 |
| 55-59 | 2008 | 102 | 9.40 | 0.93 |
| 55-59 | 2009 | 87 | 7.81 | 0.84 |
| 55-59 | 2010 | 111 | 9.67 | 0.92 |
| 55-59 | 2011 | 107 | 9.07 | 0.88 |
| 55-59 | 2012 | 104 | 8.52 | 0.84 |
| 55-59 | 2013 | 119 | 9.45 | 0.87 |
| 55-59 | 2014 | 121 | 9.39 | 0.85 |
| 55-59 | 2015 | 126 | 9.59 | 0.85 |
| 55-59 | 2016 | 111 | 8.28 | 0.79 |
| 55-59 | 2017 | 117 | 8.63 | 0.80 |
| 55-59 | 2018 | 117 | 8.53 | 0.79 |
| 55-59 | 2019 | 119 | 8.61 | 0.79 |
| 55-59 | 2020 | 95 | 6.89 | 0.71 |
| 55-59 | 2021 | 88 | 6.51 | 0.69 |
| 55-59 | 2022 | 97 | 7.34 | 0.75 |
| 55-59 | 2023 | 87 | 6.75 | 0.72 |
| 55-59 | 2024 | 72 | 5.69 | 0.67 |
| 60-64 | 2000 | 32 | 5.00 | 0.88 |
| 60-64 | 2001 | 39 | 5.93 | 0.95 |
| 60-64 | 2002 | 47 | 6.88 | 1.00 |
| 60-64 | 2003 | 35 | 4.90 | 0.83 |
| 60-64 | 2004 | 48 | 6.43 | 0.93 |
| 60-64 | 2005 | 38 | 4.88 | 0.79 |
| 60-64 | 2006 | 35 | 4.30 | 0.73 |
| 60-64 | 2007 | 52 | 5.97 | 0.83 |
| 60-64 | 2008 | 75 | 8.16 | 0.94 |
| 60-64 | 2009 | 63 | 6.56 | 0.83 |
| 60-64 | 2010 | 70 | 6.98 | 0.83 |
| 60-64 | 2011 | 65 | 6.27 | 0.78 |
| 60-64 | 2012 | 70 | 6.69 | 0.80 |
| 60-64 | 2013 | 66 | 6.17 | 0.76 |
| 60-64 | 2014 | 90 | 8.17 | 0.86 |
| 60-64 | 2015 | 74 | 6.49 | 0.75 |
| 60-64 | 2016 | 105 | 8.92 | 0.87 |
| 60-64 | 2017 | 104 | 8.56 | 0.84 |
| 60-64 | 2018 | 92 | 7.36 | 0.77 |
| 60-64 | 2019 | 97 | 7.59 | 0.77 |
| 60-64 | 2020 | 78 | 6.00 | 0.68 |
| 60-64 | 2021 | 85 | 6.42 | 0.70 |
| 60-64 | 2022 | 82 | 6.12 | 0.68 |
| 60-64 | 2023 | 86 | 6.33 | 0.68 |
| 60-64 | 2024 | 75 | 5.46 | 0.63 |
| 65-69 | 2000 | 33 | 5.57 | 0.97 |
| 65-69 | 2001 | 33 | 5.58 | 0.97 |
| 65-69 | 2002 | 22 | 3.71 | 0.79 |
| 65-69 | 2003 | 33 | 5.53 | 0.96 |
| 65-69 | 2004 | 39 | 6.41 | 1.03 |
| 65-69 | 2005 | 33 | 5.32 | 0.93 |
| 65-69 | 2006 | 43 | 6.72 | 1.02 |
| 65-69 | 2007 | 29 | 4.36 | 0.81 |
| 65-69 | 2008 | 31 | 4.46 | 0.80 |
| 65-69 | 2009 | 26 | 3.58 | 0.70 |
| 65-69 | 2010 | 36 | 4.77 | 0.80 |
| 65-69 | 2011 | 34 | 4.32 | 0.74 |
| 65-69 | 2012 | 43 | 5.10 | 0.78 |
| 65-69 | 2013 | 40 | 4.48 | 0.71 |
| 65-69 | 2014 | 54 | 5.78 | 0.79 |
| 65-69 | 2015 | 54 | 5.53 | 0.75 |
| 65-69 | 2016 | 58 | 5.73 | 0.75 |
| 65-69 | 2017 | 58 | 5.66 | 0.74 |
| 65-69 | 2018 | 61 | 5.84 | 0.75 |
| 65-69 | 2019 | 59 | 5.48 | 0.71 |
| 65-69 | 2020 | 43 | 3.86 | 0.59 |
| 65-69 | 2021 | 51 | 4.45 | 0.62 |
| 65-69 | 2022 | 57 | 4.80 | 0.64 |
| 65-69 | 2023 | 50 | 4.08 | 0.58 |
| 65-69 | 2024 | 56 | 4.46 | 0.60 |
| 70-74 | 2000 | 23 | 4.21 | 0.88 |
| 70-74 | 2001 | 26 | 4.73 | 0.93 |
| 70-74 | 2002 | 17 | 3.07 | 0.74 |
| 70-74 | 2003 | 19 | 3.42 | 0.78 |
| 70-74 | 2004 | 25 | 4.50 | 0.90 |
| 70-74 | 2005 | 16 | 2.88 | 0.72 |
| 70-74 | 2006 | 35 | 6.29 | 1.06 |
| 70-74 | 2007 | 27 | 4.84 | 0.93 |
| 70-74 | 2008 | 24 | 4.26 | 0.87 |
| 70-74 | 2009 | 18 | 3.12 | 0.74 |
| 70-74 | 2010 | 21 | 3.56 | 0.78 |
| 70-74 | 2011 | 20 | 3.28 | 0.73 |
| 70-74 | 2012 | 26 | 4.13 | 0.81 |
| 70-74 | 2013 | 30 | 4.57 | 0.83 |
| 70-74 | 2014 | 29 | 4.25 | 0.79 |
| 70-74 | 2015 | 31 | 4.38 | 0.79 |
| 70-74 | 2016 | 44 | 5.95 | 0.90 |
| 70-74 | 2017 | 21 | 2.64 | 0.58 |
| 70-74 | 2018 | 47 | 5.57 | 0.81 |
| 70-74 | 2019 | 37 | 4.17 | 0.69 |
| 70-74 | 2020 | 50 | 5.39 | 0.76 |
| 70-74 | 2021 | 37 | 3.85 | 0.63 |
| 70-74 | 2022 | 35 | 3.59 | 0.61 |
| 70-74 | 2023 | 34 | 3.41 | 0.58 |
| 70-74 | 2024 | 34 | 3.29 | 0.56 |
| 75-79 | 2000 | 16 | 3.40 | 0.85 |
| 75-79 | 2001 | 21 | 4.41 | 0.96 |
| 75-79 | 2002 | 11 | 2.30 | 0.69 |
| 75-79 | 2003 | 16 | 3.32 | 0.83 |
| 75-79 | 2004 | 18 | 3.73 | 0.88 |
| 75-79 | 2005 | 27 | 5.55 | 1.07 |
| 75-79 | 2006 | 22 | 4.49 | 0.96 |
| 75-79 | 2007 | 21 | 4.24 | 0.93 |
| 75-79 | 2008 | 11 | 2.21 | 0.67 |
| 75-79 | 2009 | 14 | 2.80 | 0.75 |
| 75-79 | 2010 | 24 | 4.78 | 0.98 |
| 75-79 | 2011 | 23 | 4.57 | 0.95 |
| 75-79 | 2012 | 19 | 3.76 | 0.86 |
| 75-79 | 2013 | 20 | 3.92 | 0.88 |
| 75-79 | 2014 | 20 | 3.84 | 0.86 |
| 75-79 | 2015 | 38 | 7.16 | 1.16 |
| 75-79 | 2016 | 32 | 5.86 | 1.04 |
| 75-79 | 2017 | 26 | 4.58 | 0.90 |
| 75-79 | 2018 | 23 | 3.88 | 0.81 |
| 75-79 | 2019 | 21 | 3.39 | 0.74 |
| 75-79 | 2020 | 27 | 4.19 | 0.81 |
| 75-79 | 2021 | 29 | 4.30 | 0.80 |
| 75-79 | 2022 | 32 | 4.40 | 0.78 |
| 75-79 | 2023 | 28 | 3.62 | 0.68 |
| 75-79 | 2024 | 33 | 4.05 | 0.70 |
| 80-84 | 2000 | 9 | 2.89 | 0.96 |
| 80-84 | 2001 | 9 | 2.75 | 0.92 |
| 80-84 | 2002 | 12 | 3.48 | 1.00 |
| 80-84 | 2003 | 9 | 2.49 | 0.83 |
| 80-84 | 2004 | 15 | 3.98 | 1.03 |
| 80-84 | 2005 | 19 | 4.93 | 1.13 |
| 80-84 | 2006 | 16 | 4.09 | 1.02 |
| 80-84 | 2007 | 15 | 3.81 | 0.98 |
| 80-84 | 2008 | 9 | 2.27 | 0.76 |
| 80-84 | 2009 | 19 | 4.76 | 1.09 |
| 80-84 | 2010 | 6 | 1.48 | 0.61 |
| 80-84 | 2011 | 17 | 4.14 | 1.01 |
| 80-84 | 2012 | 9 | 2.17 | 0.72 |
| 80-84 | 2013 | 20 | 4.80 | 1.07 |
| 80-84 | 2014 | 14 | 3.35 | 0.90 |
| 80-84 | 2015 | 14 | 3.35 | 0.90 |
| 80-84 | 2016 | 14 | 3.34 | 0.89 |
| 80-84 | 2017 | 20 | 4.74 | 1.06 |
| 80-84 | 2018 | 12 | 2.80 | 0.81 |
| 80-84 | 2019 | 21 | 4.76 | 1.04 |
| 80-84 | 2020 | 20 | 4.41 | 0.99 |
| 80-84 | 2021 | 13 | 2.77 | 0.77 |
| 80-84 | 2022 | 25 | 5.13 | 1.03 |
| 80-84 | 2023 | 19 | 3.75 | 0.86 |
| 80-84 | 2024 | 14 | 2.64 | 0.71 |
| 85+ | 2000 | 5 | 1.76 | 0.79 |
| 85+ | 2001 | 7 | 2.39 | 0.90 |
| 85+ | 2002 | 8 | 2.64 | 0.93 |
| 85+ | 2003 | 19 | 6.11 | 1.40 |
| 85+ | 2004 | 12 | 3.75 | 1.08 |
| 85+ | 2005 | 12 | 3.57 | 1.03 |
| 85+ | 2006 | 8 | 2.24 | 0.79 |
| 85+ | 2007 | 17 | 4.54 | 1.10 |
| 85+ | 2008 | 18 | 4.60 | 1.08 |
| 85+ | 2009 | 14 | 3.42 | 0.91 |
| 85+ | 2010 | 19 | 4.45 | 1.02 |
| 85+ | 2011 | 17 | 3.85 | 0.93 |
| 85+ | 2012 | 17 | 3.73 | 0.90 |
| 85+ | 2013 | 25 | 5.34 | 1.07 |
| 85+ | 2014 | 20 | 4.18 | 0.93 |
| 85+ | 2015 | 15 | 3.07 | 0.79 |
| 85+ | 2016 | 23 | 4.58 | 0.96 |
| 85+ | 2017 | 23 | 4.49 | 0.94 |
| 85+ | 2018 | 22 | 4.22 | 0.90 |
| 85+ | 2019 | 13 | 2.46 | 0.68 |
| 85+ | 2020 | 21 | 3.92 | 0.86 |
| 85+ | 2021 | 19 | 3.48 | 0.80 |
| 85+ | 2022 | 5 | 2.17 | 0.63 |
| 85+ | 2023 | 10 | 2.67 | 0.69 |
| 85+ | 2024 | 17 | 4.52 | 0.89 |
