# Supplementary material for: Trends in Canadian Suicide Mortality by Age and Sex From 2000 to 2024: Tendances en matière de mortalité attribuable au suicide au Canada selon l’âge et le sexe, de 2000 à 2024
Source: Can J Psychiatry. 2026 Sep 3:07067437261480922. Online ahead of print. doi: 10.1177/07067437261480922 (PMC13541989; doi:10.1177/07067437261480922)
Supplement: Supplementary file 5 [file sj-docx-5-cpa-10.1177_07067437261480922.docx]

**Appendix E.** Joinpoint regression results: annual percent change (APC).

Table E.1. APC metrics for overall (all ages, both sexes) and sex-stratified groups

|  | Segment Start | Segment End | APC | 95% LCL | 95% UCL | P-Value |
| --- | --- | --- | --- | --- | --- | --- |
| Overall | | | | | | |
|  | 2000 | 2007 | -1.181 | -4.3727 | -0.12 | 0.027 |
|  | 2007 | 2018 | 1.0887 | 0.5535 | 3.0812 | 0.0074 |
|  | 2018 | 2024 | -4.8528 | -6.2614 | -3.675 | 0.0024 |
| Males | | | | | | |
|  | 2000 | 2007 | -1.6164 | -4.6935 | -0.509 | 0.0048 |
|  | 2007 | 2018 | 0.9382 | 0.3833 | 3.0461 | 0.004 |
|  | 2018 | 2024 | -4.9017 | -6.4665 | -3.653 | 0.001 |
| Females | | | | | | |
|  | 2000 | 2012 | 0.6082 | -1.2587 | 1.1489 | 0.2414 |
|  | 2012 | 2016 | 3.935 | 1.4135 | 6.3584 | 0.0298 |
|  | 2016 | 2024 | -3.8889 | -4.8981 | -3.0193 | 0.007 |

Table E.2. APC metrics for five-year age by sex stratifications

|  | Segment Start | Segment End | APC | 95% LCL | 95% UCL | P-Value |
| --- | --- | --- | --- | --- | --- | --- |
| Males | | | | | | |
| 10-14 | 2000 | 2024 | -1.448 | -2.9134 | -0.0512 | 0.0434 |
| 15-19 | 2000 | 2006 | -5.65 | -17.0183 | -1.3781 | 0.012 |
| 15-19 | 2006 | 2018 | 1.6575 | -0.0266 | 12.4282 | 0.0532 |
| 15-19 | 2018 | 2024 | -8.3738 | -17.8565 | -3.5176 | 0.0112 |
| 20-24 | 2000 | 2014 | -2.3336 | -4.7285 | -1.354 | 0.0022 |
| 20-24 | 2014 | 2018 | 8.2687 | 1.9142 | 14.6943 | 0.005 |
| 20-24 | 2018 | 2024 | -10.1828 | -14.3069 | -7.7649 | 0.0008 |
| 25-29 | 2000 | 2010 | -2.6046 | -5.0678 | -1.245 | 0 |
| 25-29 | 2010 | 2019 | 3.1426 | 1.575 | 7.6741 | 0 |
| 25-29 | 2019 | 2024 | -9.687 | -13.8206 | -6.6693 | 0 |
| 30-34 | 2000 | 2007 | -4.0598 | -9.0089 | -1.7799 | 0.0018 |
| 30-34 | 2007 | 2020 | 1.2143 | 0.3287 | 6.3873 | 0.0088 |
| 30-34 | 2020 | 2024 | -7.0213 | -14.8449 | -2.4244 | 0.0016 |
| 35-39 | 2000 | 2012 | -2.1862 | -6.0603 | -1.2568 | 0.0228 |
| 35-39 | 2012 | 2022 | 0.5068 | -0.6249 | 6.5885 | 0.2988 |
| 35-39 | 2022 | 2024 | -13.4434 | -21.3297 | -3.6692 | 0.0032 |
| 40-44 | 2000 | 2024 | -1.1922 | -1.6845 | -0.728 | 0 |
| 45-49 | 2000 | 2018 | -0.0125 | -0.725 | 3.6372 | 0.919 |
| 45-49 | 2018 | 2024 | -5.6724 | -17.8015 | -1.673 | 0.0006 |
| 50-54 | 2000 | 2017 | 0.3093 | -0.2739 | 1.4848 | 0.2694 |
| 50-54 | 2017 | 2024 | -3.8719 | -9.5347 | -1.7016 | 0 |
| 55-59 | 2000 | 2018 | 1.2447 | 0.6451 | 2.2977 | 0 |
| 55-59 | 2018 | 2024 | -4.1598 | -10.0987 | -1.5274 | 0.003 |
| 60-64 | 2000 | 2019 | 1.3273 | -0.3352 | 29.4246 | 0.0602 |
| 60-64 | 2019 | 2024 | -3.4698 | -18.4705 | 1.2313 | 0.1914 |
| 65-69 | 2000 | 2017 | 1.1022 | 0.2341 | 14.0764 | 0.0288 |
| 65-69 | 2017 | 2024 | -2.5458 | -13.1161 | 0.1011 | 0.0612 |
| 70-74 | 2000 | 2024 | -0.8767 | -1.5595 | -0.0776 | 0.0338 |
| 75-79 | 2000 | 2018 | -0.4294 | -1.4608 | 9.9679 | 0.689 |
| 75-79 | 2018 | 2024 | -6.808 | -21.156 | -2.0388 | 0.002 |
| 80-84 | 2000 | 2015 | 1.923 | 0.3906 | 6.5464 | 0.0176 |
| 80-84 | 2015 | 2024 | -5.1188 | -12.407 | -2.136 | 0.0002 |
| 85+ | 2000 | 2020 | -0.3288 | -1.2079 | 4.9515 | 0.84 |
| 85+ | 2020 | 2024 | -10.5322 | -26.0281 | -2.2518 | 0.0022 |
| Females | | | | | | |
| 10-14 | 2000 | 2024 | 2.4032 | 0.7218 | 4.3947 | 0.007 |
| 15-19 | 2000 | 2022 | 1.9069 | 1.24 | 3.0288 | 0.0006 |
| 15-19 | 2022 | 2024 | -21.6546 | -33.4093 | -3.9098 | 0.0164 |
| 20-24 | 2000 | 2006 | -2.6895 | -16.306 | 3.3851 | 0.337 |
| 20-24 | 2006 | 2018 | 4.4157 | -6.7803 | 19.3857 | 0.0612 |
| 20-24 | 2018 | 2024 | -3.884 | -14.0732 | 3.046 | 0.1262 |
| 25-29 | 2000 | 2024 | 1.8503 | 1.0022 | 2.8475 | 0 |
| 30-34 | 2000 | 2024 | 0.5649 | -0.2823 | 1.4927 | 0.1686 |
| 35-39 | 2000 | 2024 | -0.4776 | -1.1385 | 0.1855 | 0.1502 |
| 40-44 | 2000 | 2022 | -0.3576 | -0.9534 | 1.2605 | 0.4412 |
| 40-44 | 2022 | 2024 | -18.7778 | -29.9498 | -1.924 | 0.0034 |
| 45-49 | 2000 | 2016 | -0.1505 | -1.0987 | 4.2887 | 0.934 |
| 45-49 | 2016 | 2024 | -4.8129 | -16.7458 | -1.9822 | 0 |
| 50-54 | 2000 | 2018 | 0.8853 | 0.0022 | 2.3346 | 0.0498 |
| 50-54 | 2018 | 2024 | -8.445 | -18.017 | -4.1629 | 0 |
| 55-59 | 2000 | 2014 | 2.7281 | 1.4803 | 5.024 | 0 |
| 55-59 | 2014 | 2024 | -4.322 | -7.5025 | -2.4982 | 0 |
| 60-64 | 2000 | 2017 | 2.4757 | 1.2215 | 7.6732 | 0 |
| 60-64 | 2017 | 2024 | -5.0986 | -16.8256 | -0.8063 | 0.0206 |
| 65-69 | 2000 | 2024 | -0.6097 | -1.7256 | 0.8051 | 0.3996 |
| 70-74 | 2000 | 2024 | -0.1792 | -1.5799 | 1.4733 | 0.9336 |
| 75-79 | 2000 | 2024 | 0.4864 | -0.9698 | 2.2103 | 0.4524 |
| 80-84 | 2000 | 2024 | 0.5509 | -1.3953 | 2.8672 | 0.5034 |
| 85+ | 2000 | 2003 | 39.6525 | 0.0221 | 200.9478 | 0.0498 |
| 85+ | 2003 | 2024 | -1.1536 | -14.3293 | 1.0416 | 0.1902 |
